# Novel Immune biomarkers for the early stratification of oligoarthritis patients at risk of developing polyarticular extension

**DOI:** 10.3389/fimmu.2025.1663663

**Published:** 2025-10-08

**Authors:** Federica Raggi, Simone Pelassa, Francesca Antonini, Chiara Rossi, Federica Briasco, Silvia Maria Orsi, Genny Del Zotto, Davide Cangelosi, Angelo Ravelli, Marco Gattorno, Alessandro Consolaro, Maria Carla Bosco

**Affiliations:** ^1^ Unit of Rheumatology and Autoinflammatory Diseases, Department of Pediatric Sciences, Istituto di Ricovero e Cura a Carattere Scientifico (IRCCS) Istituto Giannina Gaslini, Genova, Italy; ^2^ Core facilities Laboratory, Integrated Department of Services and Laboratories, IRCCS Istituto Giannina Gaslini, Genova, Italy; ^3^ Department of Neuroscience, Rehabilitation, Ophthalmology, Genetics, Maternal and Child Health, University of Genova, Genova, Italy; ^4^ Clinical Bionformatics Unit, IRCCS Istituto Giannina Gaslini, Genova, Italy; ^5^ Scientific Direction, IRCCS Istituto Giannina Gaslini, Genova, Italy

**Keywords:** juvenile idiopathic arthritis (JIA), oligoarthritis, polyarticular extension, biomarkers, Tcells/monocyte/macrophages, extracellular vesicles (EVs), immune signature

## Abstract

**Introduction:**

Oligoarthritis, the most common form of Juvenile Idiopathic Arthritis in Western countries and a leading cause of disability, exhibits a variable clinical course. Early identification of children at risk of polyarticular extension is critical for guiding targeted therapy, but requires new biomarkers. This study aimed at profiling T cell and monocyte/macrophage (MM) subset composition and activation/maturation state combined with extracellular vesicle (EV) surface markers in synovial fluid (SF) and peripheral blood (PB) from new-onset Oligoarthritis patients to prospectively evaluate their correlation with clinical course over a two-year follow-up period and identify potential prognostic biomarkers.

**Methods:**

SF and PB samples were collected from 42 untreated patients at disease onset. Immune cell subsets were analyzed by flow cytometry, EV marker expression profiles by bead-based multiplex assays, and soluble TREM1 (sTREM1) levels by ELLA. Differences between patients exhibiting oligoarticular course (Group 1) or polyarticular extension (Group 2) over two years of follow-up were assessed.

**Results:**

Group 2 patients showed significantly higher CD3:CD14 ratio (AUC = 0.831,p<0.005) and HLA-DR+ CD4+ T cell percentages (64.8%vs52.5%,p=0.02) in SF compared to Group 1 patients. In PB, both HLA-DR+CD4+ and HLA-DR+CD8+ cells were significantly increased (AUC = 0.946,p<0.001) in Group 2. Group 2 patients also exhibited significantly higher proportions of effector memory (EM) CD4+ (AUC = 0.911, p<0.001) and CD8+ (AUC:0.929, p<0.001) subsets, along with lower proportions of naïve CD4+ (AUC = 0.929, p<0.001) and CD8+ (AUC = 0.893, p<0.001) subsets in the circulation, that was reflected in a significantly higher EM:naïve ratios for both CD4+ (AUC = 0.893,p<0.001) and CD8+ (AUC = 0.946;p<0.001) populations. TREM1+ CD14+ cell percentages in both SF and PB were significantly (p<0.05) lower (SF: 83.6%vs90.47%; PB:40.16%vs53.21%), while sTREM1 levels higher (SF: 8926vs5822 pg/ml; PB:298.8vs232 pg/ml), in Group 2 compared to Group 1. Finally, SF-derived EVs from Group 2 showed significantly reduced HLA-ABC (AUC = 0.857,p=0.012) and CD3 (AUC = 0.949,p<0.001) expression. Combining these markers further improved the discriminatory performance of the models (AUC = 1,p<0.001).

**Discussion:**

This exploratory study identifies novel immune classifiers combining T lymphocytes and MM subsets with EV markers which stratify, at onset, Oligoarthritis patients who will develop polyarticular extension and provide important mechanistic insights into arthritis progression.

## Introduction

Oligoarthritis is the most common form of Juvenile Idiopathic Arthritis (JIA) in many geographical areas, especially Western countries (40-60%), and a major cause of disability in children (1–4). The disease is characterized by early arthritis onset in ≤4 joints, female predominance, asymmetry, high frequency of antinuclear antibodies (ANA), elevated incidence of chronic uveitis (15-30% of cases), and a strong association with HLA-DRB1*0801 (12-30% of cases) (2, 5, 6). Children with Oligoarthritis can have variable clinical courses and therapeutic outcomes (7). Most patients experience a benign oligoarticular course with disease confined to ≤4 joints, often achieving remission in response to intra-articular corticosteroids (8). However, a significant proportion of children (21-40%) present a less favorable outcome, characterized by a more severe, erosive disease course with arthritis extending to ≥4 joints within two years, ultimately resulting in structural damage and functional impairment (9–11). These patients may not respond adequately to intra-articular steroids (2, 12, 13) and require a more aggressive therapeutic intervention with synthetic or biologic disease-modifying anti-rheumatic drugs (DMARDs) (8, 10, 13–16). Risk stratification is essential to identify children at higher risk of polyarticular extension early in the disease process to enable more tailored treatment strategies aimed at achieving an inactive status or, at least, minimal activity at an earlier stage, thereby reducing disease burden, minimizing the side effects of ineffective medications, and enhancing patient quality of life (2, 8, 14, 17, 18). Many efforts over the past decades have attempted to identify novel early predictors of disease course in Oligoarthritis patients (17–22). Although several clinical indicators of disease extension have been proposed (9, 10, 23, 24), no validated prognostic biomarkers have yet been incorporated into routine clinical practice.

Cells of both innate and adaptive immunity are enriched in the synovial fluid (SF) of active joints in Oligoarthritis patients, playing a key role in joint inflammation and cartilage degradation, either directly and/or by activating resident synovial cells (25–29). Studies analyzing the SF infiltrate have identified differences in lymphocyte subsets that correlated with disease outcomes (7, 19, 30, 31). Proinflammatory interleukin-17-producing T cells (Th17) were observed in children who developed a polyarticular outcome (32), while higher levels of immunoregulatory T-cells (Treg) were seen in patients who retained the oligoarticular phenotype (33–35), with a direct reciprocal relationship between the two T-cell populations within the same joints (26, 32). Additionally, higher percentages of activated memory B cells and lower percentages of naive B cells were found in the SF of Oligoarthritis patients at relapse compared to disease onset (36). These findings raised the possibility that the composition of synovial lymphocytes at disease onset may represent a potential early predictor of polyarticular extension (36, 37). However, this hypothesis has not been experimentally tested further. In addition, although recent studies have reported differences in T and B cell subset in the peripheral blood (PB) of Oligoarthritis patients with different outcome (30), to our knowledge no study has specifically analyzed the correlation between PB immune cell subset composition at disease onset and patient clinical course at follow up, which could lead to easily measurable, non-invasive biomarkers.

Immune cells, in particular T lymphocytes and monocytes/macrophages (MMs), infiltrating the joints of patients with rheumatic diseases actively release extracellular vesicles (EVs) (38, 39). These heterogeneous nanosize particles, delimited by lipid bilayer membranes, are important mediators of cell-to-cell communication and exert key roles in antigen presentation, immune regulation, and inflammation due to their cargo of bioactive molecules of cellular origin (nucleic acids, proteins, and lipids), which can be transferred to, and elicit responses in, recipient cells (40–44). In recent years, EVs have been implicated in the pathogenesis of various chronic inflammatory and autoimmune disorders (45–47), including arthritides (38, 48–50), by promoting pro-inflammatory and tissue-destructive cellular responses (38, 40, 41, 51). In addition, EVs are increasingly recognized as a promising source of novel diagnostic/prognostic biomarkers for these conditions (5, 52–54). We have recently demonstrated that EV miRNomic and proteomic profiles in the SF and plasma (PL) of Oligoarthritis patients could provide new early putative molecular indicators of disease development and progression (5, 39, 55). However, the characterization of EV surface protein cargo and of its potential value as a biomarker for Oligoarthritis remains unexplored, despite such an approach has been successfully applied in other diseases (56–59).

This study was designed as an exploratory investigation to identify novel early candidate prognostic immune biomarkers in Oligoarthritis by phenotyping T cell and MM subsets in SF and PB samples and profiling SF-derived EV surface markers in a cohort of treatment-naïve patients at disease onset and by prospectively evaluating their correlation with clinical course over a two-year follow-up period.

## Materials and methods

### Study population

New-onset Oligoarthritis children (age 1–16 years) meeting the 2001 International League for Associations of Rheumatology (ILAR) classification criteria (involvement of ≤4 joints in the first 6 months of disease) (60) and undergoing therapeutic arthrocentesis were recruited at the time of diagnosis at the IRCCS Giannina Gaslini Institute, Genova, between 2018 and 2021, and followed up for 2 years at a 3-month interval. All patients had clinically active disease, with joint effusion, swelling, pain, and stiffness at the time of sampling, and onset of symptoms for more than 6 weeks and no more than 6 months before enrollment. The number of active joints at disease onset was determined by standard clinical evaluation associated with ultrasound or magnetic resonance imaging. Various clinical and laboratory parameters and known markers of disease activity, such as ANA, C-reactive protein (CRP), and erythrocyte sedimentation rate (ESR) (9), were measured. A previous or current treatment with synthetic or biologic anti-inflammatory drugs at the time of disease presentation was considered as an exclusion criterion. All enrolled patients underwent arthrocentesis at diagnosis and received IAS treatment, either as monotherapy or in combination with MTX and/or biologic agents. Different therapeutic regimens were administered during the follow-up period. Disease relapse (referred to as a flare-up in the joints involved at diagnosis and/or appearance in other joints), maintenance of the oligoarticular course or progression to polyarticular extension, and iridocyclitis development were determined prospectively during the follow-up evaluation. The study protocol was reviewed and approved by the Ethics Committee of the Region Liguria (Approval 165/2018), and the procedures were carried out according to the approved guidelines and in adherence to the general ethical principles set forth in the Declaration of Helsinki. Written informed consent to participate in the study was obtained from the parents or the patient’s legal guardian prior to sample collection.

### Sample collection

SF aspirates were obtained at disease onset by knee arthrocentesis and collected into tubes containing EDTA. Arthrocentesis was performed under local anesthesia or, in case of younger patients or multiple joints, under general sedation. Paired PB samples were collected by venipuncture into EDTA tubes using vacutainer systems. Specimens were centrifuged at 500 x g for 10 minutes at room temperature (RT) to obtain cell-free SF and plasma (PL) and stored at -80 °C until use. PB- and SF-derived mononuclear cells (PBMCs and SFMCs) were isolated by sample centrifugation on a Ficoll gradient as described (61, 62) and stored in liquid nitrogen until analysis.

### Flow cytometry characterization of PBMCs and SFMCs

PBMCs and SFMCs were stained with different combinations of fluorochrome-conjugated monoclonal antibodies (mAbs) directed against surface antigens and activation/polarization markers of T lymphocytes, such as T helper/cytotoxic, naïve/central memory/effector-memory/effector-memory RA and Treg (CD3, CD4, CD8, CD45RO, CD45RA, CCR7, HLA-DR, CD25, CD127), as well as classical M1- and alternative M2- polarized MM subsets (CD14, CD80, CD206, CD163, and the triggering-receptor expressed on myeloid cells TREM-1). Stained cells were analyzed using a Flow cytometer FACSFortessa (BD, Mountain View, CA, USA) and the Kaluza 2.1 program. Briefly, cells were resuspended with the Brilliant Stain Buffer (BD, Milano, Italy) and incubated with mouse anti-human mAbs for 30 min at 4°C, after blocking nonspecific sites by preincubation with FCR Blocking Reagent (MACS Miltenyi Biotec). The mAbs used are listed in **Supplementary Table S1**. Cells were gated according to their light-scatter properties to exclude cell debris. The main cell populations were identified using sequential gating strategy after doublet exclusion.

### viSNE and FLowSOM analysis

The Cytobank platform was employed to conduct a dimensionality reduction analysis of T cell flow cytometry data using viSNE (63). viSNE analysis transformed FACS data by applying the t-Distributed Stochastic Neighbor Embedding (t-SNE) algorithm to arrange cells in a 2D map, thus representing phenotypic similarity based on markers measured by flow cytometry. This approach provided a visual representation of single-cell data similar to a biaxial plot, where each cell position reflects its proximity to others in high-dimensional space. The CD3, CD4, CD8, CD45RO, CD45RA, CCR7 and HLA-DR markers were used as input channels for T cell viSNE analysis. A third axis (third dimension) was used to visualize HLA-DR expression levels in these cells. To identify and characterize distinct cellular subpopulations, clustering analysis was performed on the viSNE data using FlowSOM (64).

### EV isolation and evaluation of antigen expression by MACSPlex

EVs were isolated from 1 ml of SF samples using the EV Isolation Kit Pan (Miltenyi Biotec, Bologna, Italy), according to the manufacturer’s instructions (65). Before EV isolation, cell-free SF samples were treated with 2U/ml Hyaluronidase (HYase) (Sigma, Merck Life Science, Milano, Italy) for 30 minutes at 37°C to remove contaminating hyaluronan extracellular matrix (ECM) components, as detailed (39). Isolated EVs were then phenotyped using the MACSPlex Exosome Kit (Miltenyi Biotec, Bologna, Italy), a multiplex bead-based platform allowing the simultaneous detection of 37 surface epitopes and two internal negative isotype controls by EV labeling with hard-dyed capture beads coated with different PE-conjugated mAbs followed by flow cytometry (FACS) (66), according to the manufacturer instructions. In brief, EVs were resuspended in 120 µl of MACSPlex Buffer and labeled with 15 µl of MACSPlexExosomeCaptureBeads and a mix of APC-conjugated anti-CD9, anti-CD81, and anti-CD63 Abs. Samples were incubated for 1 hour at RT and then centrifuged at 3000 g for 5 minutes. A sample depleted of EVs was processed following the same protocol and used as a blank. Antigen expression was assessed using a Gallios Flow Cytometer (Beckman Coulter, Brea, California, USA) by measuring the overall MFI.

### sTREM1 release

Soluble (s)TREM-1 content was measured in PB and SF samples using the Simple Plex Human TREM-1 Cartridge (R&D Systems) and the ELLA automated microfluidic Immunoassay system. The assay range was 4.2-40,000 pg/mL and the lower limit of detection was 0.73 pg/mL. Briefly, PL and SF samples were centrifuged at 13000 g for 5 min, diluted in Sample Diluent Buffer, and then analyzed. PL samples were diluted 1:2 as suggested by the manufacturer, while SF was diluted 1:500 based on preliminary data. sTREM-1 concentrations were quantified based on an internal standard curve, according to the manufacturer instruction.

### Statistical analysis

Statistical analyses were performed using the GraphPad Prism 8.3.0 (GraphPad Software, La Jolla, CA, USA) and MedCalc software (MedCalc Software Ltd., Ostend, Belgium). Comparisons of numeric variables between the two groups of patients were performed by a two-tailed unpaired Student’s t-test or a non-parametric Mann-Whitney test after performing Shapiro-Wilk normality test. F-test was used to assess equality of variance. Comparisons of categorical variables between the two groups of patients were performed by Fisher’s exact test or Fisher-Freeman-Halton asymptotic test when distinct values were two or three, respectively. When the number of categories exceeded three, Fisher’s exact test with Monte Carlo simulation was applied to estimate the statistical association between the group and a clinical feature. Tests were implemented in the contingency tables R package version 3.0.1. Data are expressed as the median and mean ± standard error of the mean (SEM) from at least three independent experiments, unless otherwise specified. A p value of <0.05 (*), <0.01 (**), or <0.001 (***) was considered statistically significant. Receiving Operating Characteristic (ROC) Curves were plotted to visually display the discriminating power of each tested variable. The area under the ROC curve (AUC) was measured to quantitatively assess the performance of each variable. Confidence intervals (CI, 95%) were calculated for each AUC to assess stability and discriminative performance. AUC > 0,8 with p<0,05 was arbitrarily considered as indicative of a good performance, and only results meeting these criteria were reported in the paper. The combined discriminatory power of clinical, immunophenotypic variables, and EV surface markers across Group 1 and Group 2 in SF and PB was assessed by generalized linear models using the GLMNET R package (67). The group (clinical outcome) was set as the response variable, while cell subset phenotypes and EV marker expression were used as the explanatory variables. Elastic net regularization was applied with a mixing parameter (alpha) of 1.0, corresponding to LASSO regression. Model selection was based on minimizing the mean square error (MSE) with the optimal penalty parameter (lambda) identified using the leave-one-out cross-validation technique (LOOCV) implemented via the cv.glmnet function from the glmnet R package. This approach was used to minimize the risk of overfitting.

## Results

### Characteristics of the cohort of new-onset oligoarthritis patients

A cohort of forty-two patients was selected for the study and divided into two groups of twenty-one patients each, based on their clinical course over a two-year follow-up period. Group 1 included patients who maintained the oligoarticular phenotype, while Group 2 comprised those who progressed to polyarticular extension. The two-year follow-up timeframe was chosen because polyarticular extension in Oligoarthritis most commonly occurs within this period following disease onset (9). The main demographic, clinical, laboratory, and therapeutic characteristics of the two patient groups at disease onset and during follow-up are summarized in **Table 1**. The mean age at onset was comparable between groups (5.9 years in Group 1 *vs* 5.5 in Group 2), while the female-to-male ratio was slightly higher in patients of Group 2 (66,6% *vs* 52,4%). No significant association was found between sex and disease outcome (p > 0.05), as determined by Fisher’s exact tests, suggesting that this parameter is unlikely to act as a confounding variable in our analysis. In contrast, many differences were observed in clinical features. Almost sixty-two percent of children in Group 1 had involvement of a single joint at onset compared to only nineteen percent in Group 2. Conversely, over eighty percent of Group 2 patients presented arthritis in two or more joints as compared with only thirty-eight percent of patients of Group 1. The knee was the most commonly affected joint in both groups (100%). However, a higher proportion of patients in Group 2 also exhibited involvement of ankle (38% vs 19%) and small hand joint (19% vs 9.5%), as well as symmetric disease distribution (47.6% vs 14.2%), and upper limb arthritis (28.5% vs 14.2%) compared to Group 1. No differences were observed in small foot joint involvement between the two groups (9.5% vs 9.5%), and no patient presented with wrist involvement at diagnosis, regardless of their subsequent disease course. Among markers of disease activity, we observed higher mean ESR (mean 39.5 vs 23.9) and CRP (mean 1.2 vs 0.7) levels in Group 2. The frequency of ANA positivity was comparable in the two groups (87.5% vs 82.3%). Regarding treatments, intra-articular steroid injection (IAS) alone was the most commonly administered therapy immediately after diagnosis, in particular in Group 1 patient (71.4% vs 47.6%). In contrast, methotrexate (MTX), in combination with IAS, was more frequently used in Group 2 (47.6% vs 23.8%). During the follow up period, IAS continued to be more often used in Group 1 (57.1% vs 9.5%), while MTX (52.3% vs 28.6%) and other anti-inflammatory or biologic agents (38.1% vs 14.3%) were more frequently prescribed in Group 2 patients. By the end of the two-year follow-up period, all patients in Group 2 had experienced articular relapse compared to about half in Group 1 (100% vs 57%). Conversely, a greater proportion of patients of Group 1 had developed iridocyclitis (19% vs 9.5%). Statistically significant differences were observed between Group 1 and Group 2 in terms of ESR levels, number of active joints, and frequency of symmetric joint involvement at onset, incidence of articular relapse and types of treatment administered during follow-up (p<0.05).

**Table 1:** Demographic, clinical, laboratory, and therapeutic features of Oligoarthritis patients at disease onset and follow up^a^.

Patient features	Group 1	Group 2	P-value^f^
N of patients^b^	21	21	
Female^b^	11(52.4)	14 (66.6)	0,53
Age at onset (yrs)^c^	5.9 (1.6-13)	5.5 (1.1-16)	0,31
CRP (mg/dL)^c^	0.7 (0.2-3.55)	1.2 (0.2-8.7)	0,69
ESR (mm/h) ^c^	23.9 (4-75)	39.5 (7-120)	0,02
ANA positivity^b*^	14 (87.5)^£^	14 (82.3)^$^	1
No. of active joints (at sampling)^b^			0,004
One	13 (61.9)	4 (19.0)	
Two	3 (14.3)	11 (52.3)	
Three	2 (9.5)	0 (0)	
Four	3 (14.3)	6 (28.5)	
Type of involved joints^b^			0,53
Knee	21 (100)	21 (100)	
Ankle	4 (19)	8 (38)	
Small hand joints	2 (9.5)	4 (19)	
Small foot joints	2 (9.5)	2 (9.5)	
Symmetric joints^b^	3 (14.2)	10 (47.6)	0,04
Upper limbs^b^	3 (14.2)	6 (28.5)	0,45
Relapse (within 2 yrs)^b^	12 (57)	21 (100)	0,001
Iridocyclitis (within 2 yrs)^b^	4 (19)	2 (9.5)	0,66
Drug therapies (after onset)^b,d^			0,25
IAS	15 (71.4)	10 (47.6)	
MTX (+IAS)	5 (23.8)	10 (47.6)	
Biologic Treatment (+IAS ± MTX)	1 (4.8)	1 (4.7)	
Drug therapies (within 2 yrs)^b,e^			0,004
IAS	12 (57.1)	2 (9.5)	
MTX ( ± IAS)	6 (28.6)	11 (52.3)	
Biologic Treatment ( ± IAS ± MTX)	3 (14.3)	8 (38.1)	

a The table reports the main characteristics at onset of the Oligoarthritris patients analyzed in the study divided into two groups based on disease course by two years of follow-up (Group 1=oligoarticular course; Group 2=polyarticular course). Disease activity was defined by the presence of joint effusion and swelling, limitation of movement with either pain on movement or tenderness.

b Results are expressed as number of patients (percentage in parenthesis).

c Results are expressed as mean (range in parenthesis).

d Drug therapies administered after disease initial presentation.

e Drug therapies administered during the 2 years of follow-up.

f P-values obtained comparing Group 1 and Group 2 with Fischer's exact, Fischer-Freeman-Halton, Fisher's Exact Test with Monte Carlo simulation.

*Patients were defined as ANA+ positive if they had 2 positive results on indirect immunofluorescence at a titre of ≥1:160. £Available for 16 patients; $ available for 17 patients.

ESR, erithrocyte sedimentation rate; CRP, C-reactive protein; ANA, anti-nuclear antibodies; IAS, Intra-articular steroid; MTX, Methotrexate.

### The proportion and activation state of T lymphocyte and MM subsets in SF at onset can differentiate oligoarthritis patients who will follow an oligoarticular *vs* a polyarticular course

Experiments were conducted to characterize the composition of SFMCs at disease onset and identify potential differences in T cell and MM subset proportions and activation states between patients experiencing different clinical courses at follow-up. A total of twenty-eight patients (n=14 per group), selected based on the availability of samples with an adequate number of viable cells for flow cytometric analysis, were included in the analysis, This number exceeded the threshold determined through an *a priori* power analysis conducted using G*Power software (version 3.1.9.7) (68), which estimated that a minimum of 11 patients per group would be required to detect a two-fold difference between group means with a standard deviation ≤50% of the mean (α = 0.05, power (1–β) = 0.8, effect size d = 1.26), confirming sufficient power to detect meaningful differences in immune marker expression between outcome groups. Care was also taken to ensure a balanced distribution between the two clinical groups minimizing potential selection bias.

**Figure 1:**
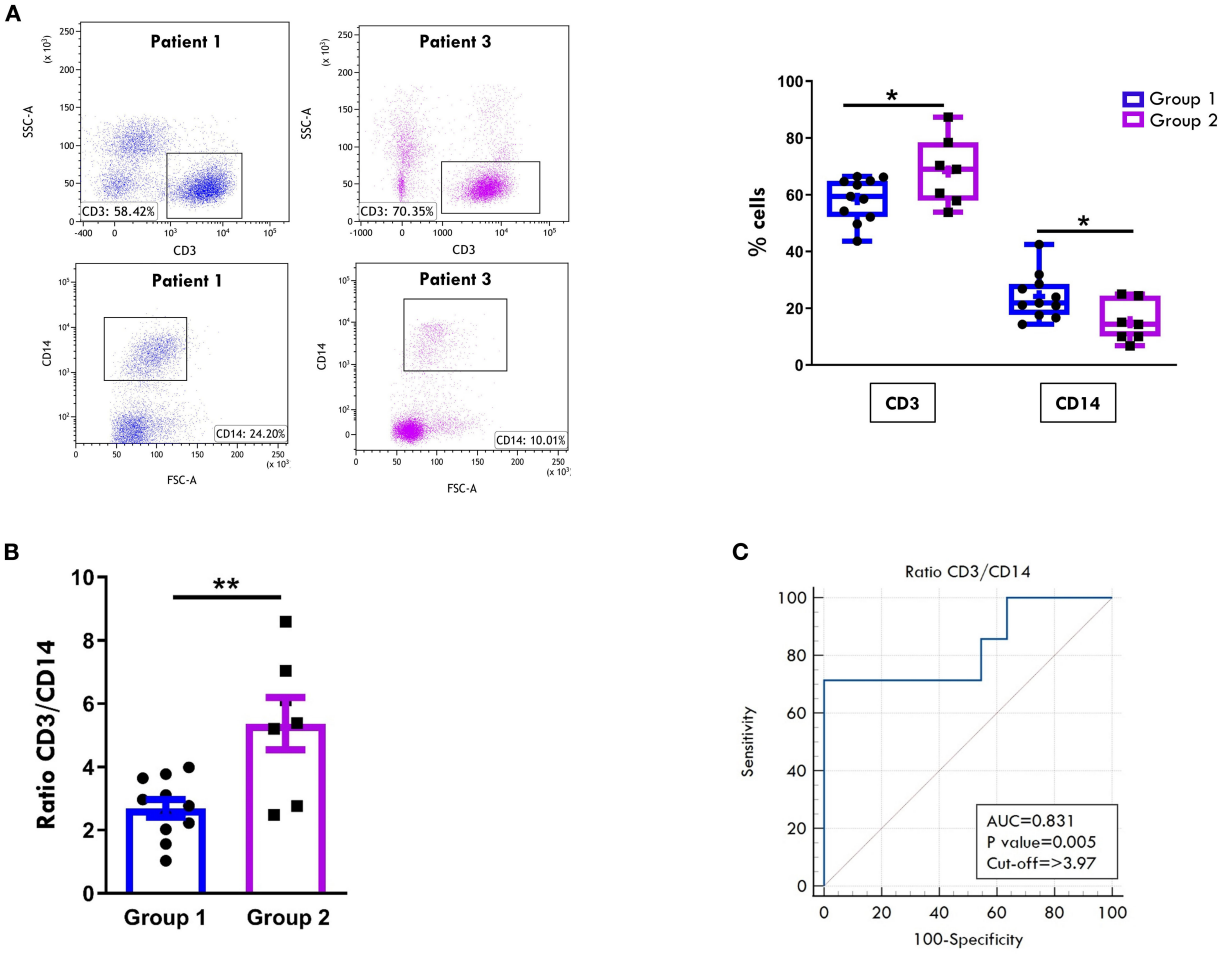
Comparative analysis of T cell and monocyte/macrophage composition in SF between patients undergoing different disease courses. SFMCs were purified from new-onset oligoarthritis patients, stained with Abs against CD3 and CD14 antigens, and analyzed by multi-color flow cytometry using a gating strategy based on light scattering properties and doublet exclusion. **(A)** Flow cytometry plots *(left*) show results of one representative patient from Group 1 (patient 1) and one from Group 2 (patient 3). The numbers within each plot indicate CD3+ and CD14+ cell percentages. Box plots (*right*) depict the median percentages of CD3+ and CD14+ cells in all tested patients of Group 1 and Group 2. Boxes comprise the values falling between the 25th and 75th percentiles, the horizontal lines within each box indicate the median values, whiskers (lines that extend from the boxes) represent the highest and lowest values for each group, the "+" symbols represent the mean values. Individual data points overlaid on the box plots represent each single patient. *P* values of CD3+ cell percentages in Group 1 relative to Group 2: *p ≤ 0.05; *P* values of CD14+ cell percentages in Group 1 relative to Group 2: *p ≤ 0. **(B)** CD3:CD14 ratio in patients from Groups 1 and 2 is presented as a bar graph. Individual data points overlaid on the bar graph represent each single patient. Results represent the means ± SEM. *P* values for the ratio of Group 1 relative to Group 2: **p ≤ 0.01. **(C)** ROC curve shows the power of the CD3:CD14 ratio in discriminating between Group 1 and Group 2. Sensitivity (%) is shown on the Y-axis, and 100% specificity (%) is shown on the X-axis. Areas under the curves (AUC), p-values, and cut-off values are reported for each graph. *P* values of CD3:CD14 ratio of Group 1 relative to Group 2: p ≤ 0.01.

Eight-color flow cytometric analysis showed an inverse relationship in the overall proportions of CD3+ T cells and CD14+ MMs between the two groups. Although both groups exhibited a predominance of CD3+ over CD14+ cell population, patients of Group 2 displayed significantly higher CD3+ (68.2% ± 4.4 vs. 58.4% ± 2.3; p= 0.049) and lower CD14+ cell percentages (15.07 ± 2.7 vs 24.19 ± 2.4; p=0.026) (**Figure 1A**). These differences were reflected in a significantly higher CD3:CD14 ratio in Group 2 (5.3 ± 0.8 vs 2.6 ± 0.2; p=0.002) (**Figure 1B**). Given that the CD3:CD14 ratio is a simple, routinely measurable laboratory parameter, its ability to discriminate between the two groups was evaluated by ROC analysis. A cut-off value of 3.97 was identified as optimal for distinguishing at onset patients who would progress to polyarticular extension with an AUC of 0.831 (p=0.005), yielding a sensitivity of 71.43% and a specificity of 100% (**Figure 1C**). These findings indicate a good discriminatory power of the CD3:CD14 ratio.

**Figure 2:**
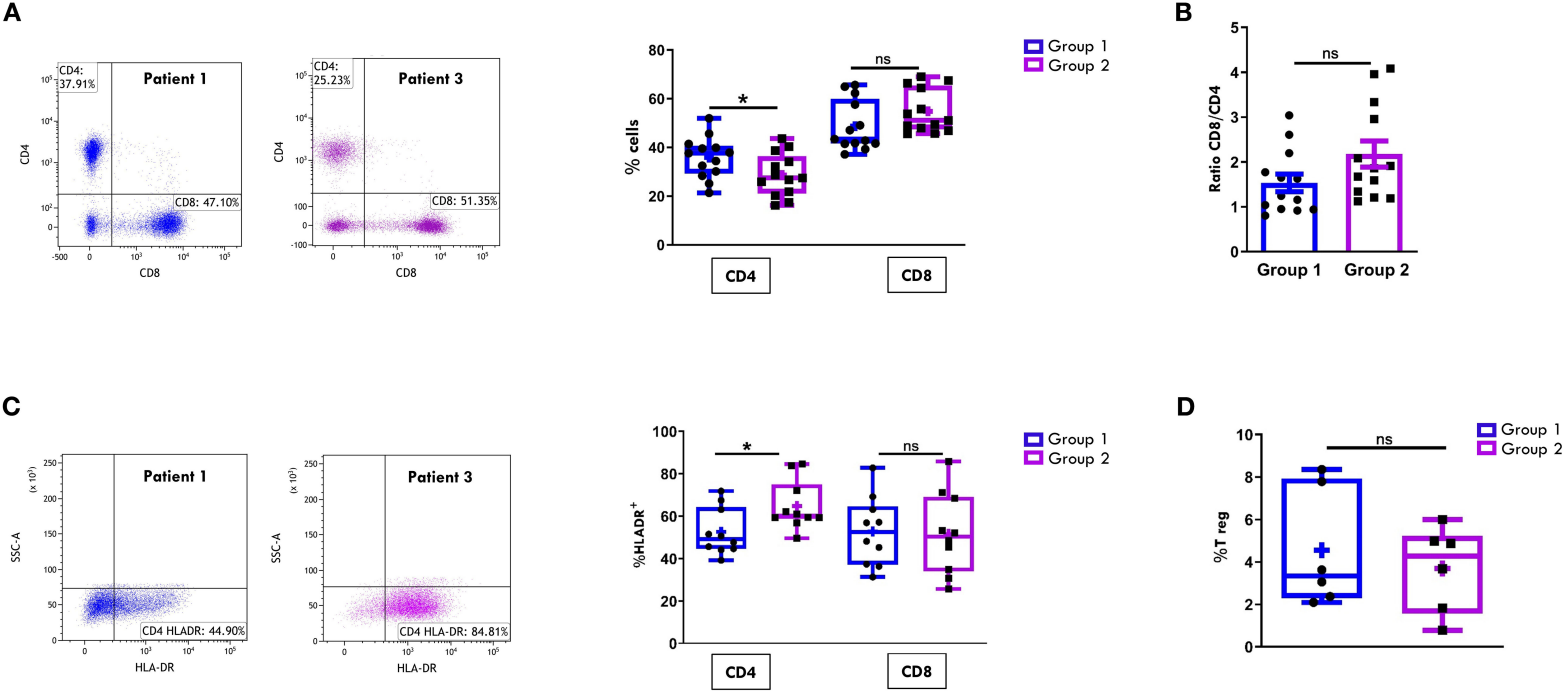
Comparative analysis of CD4/CD8 T Cell proportions and activation in SF between patients undergoing different disease courses. SFMCs were stained with Abs against CD3, CD4, CD8, HLA-DR, CD25 and/or CD127 antigens and analyzed by multicolor flow cytometry using a gating strategy based on light scattering properties and doublet exclusion. CD3-gated cells were analyzed for CD4 and CD8 positivity, and CD8+ and CD4+ cells were then examined for HLA-DR expression. **(A)** Flow cytometry plots (*left*) show CD4/CD8 positivity of one representative patient from Group 1 (patient 1) and one from Group 2 (patient 3). Box plots (*right*) depict the median percentages of CD4+ and CD8+ cells in all tested patients of Group 1 and Group 2. Results are presented as described in **Figure 1A**. *P* values of CD4 percentage in Group 1 relative to Group 2: *p ≤ 0.05; ns: not significant. **(B)** CD8:CD4 ratio in patients from Group 1 and 2 is presented as a bar graph. Results are indicated as described in **Figure 1B**. ns, not significant. **(C)** Flow cytometry plots (*left*) show HLA-DR positivity of one representative patient from Group 1 (patient 1) and one from Group 2 (patient 3). Box plots (*right*) show the median percentages of HLA-DR+ cells in CD4+ and CD8+ cell subsets. Results are presented as described in **Figure 1A**. P values for HLA-DR+ CD4+ cell percentages in Group 1 relative to Group 2: *p ≤ 0.05; ns, not significant. **(D)** Box plots show the median percentages of T Reg+ cells identified as CD4^+^CD25^+^CD127^-^ cells in tested patients of Group 1 and 2. Results are presented as in **Figure 1A**. ns, not significant.

We next compared the CD4+ and CD8+ subset proportions within the CD3+ cell population between the two groups. As shown in **Figure 2A**, all patients at disease onset exhibited a predominance of cytotoxic CD8^+^ T cells over helper CD4^+^ T cells, regardless of disease progression. However, Group 2 patients showed higher, though not statistically significant, percentages of CD8^+^ cells (54.8% ± 2.4 vs 48.7% ± 2.8) and significantly lower proportions of CD4+ cells (28.73% ± 2.4 vs 35.85% ± 2.3; p= 0.04) compared to Group 1. Accordingly, the CD8:CD4 ratio was higher in Group 2 patients (2.1 ± 0.2 vs. 1.5 ± 0.19), although this difference did not reach statistical significance (**Figure 2B**). Furthermore, patients in Group 2 exhibited a significantly higher proportion of activated cells within the CD4+ population, as indicated by HLA-DR expression (64.8% ± 3.6 vs. 52.5% ± 3.4; p value: 0.02), whereas no significant differences in HLA-DR+ proportion were observed between the two groups in the CD8+cell compartment (**Figure 2C**). To further characterize the T cell profile of SFMCs, we evaluated the proportion of the immunosuppressive regulatory T cells (Tregs), defined as CD4+CD25+/CD127- cells. As depicted in **Figure 2D**, Treg frequencies were comparable between the two groups of patients, indicating that the observed differences in CD4+ T cell activation were not attributable to alterations in the Treg compartment.

**Figure 3:**
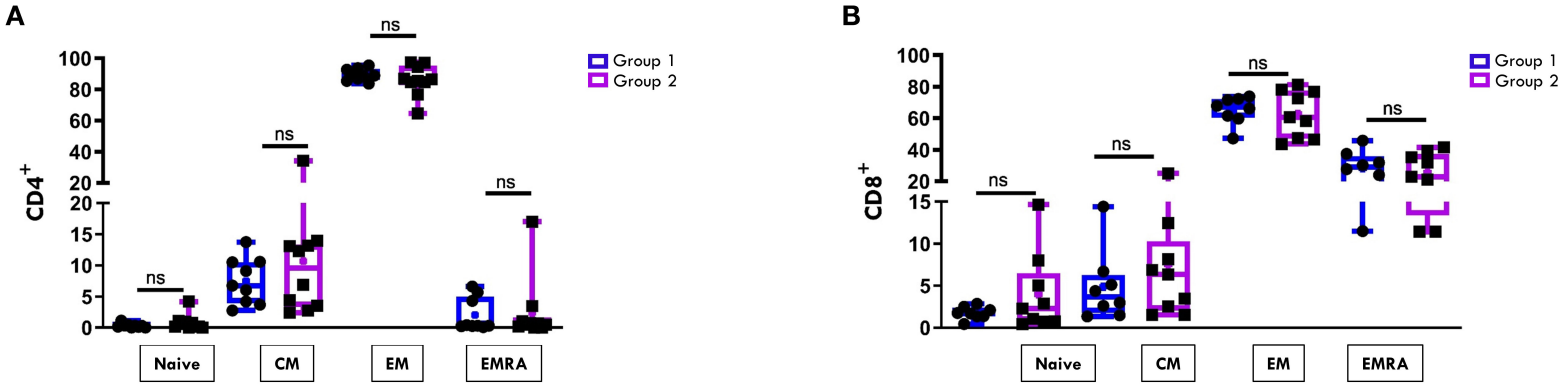
Comparative analysis of CD4/CD8 T Cell maturation subsets in SF between patients undergoing different disease courses. SFMCs were stained with Abs against CD4, CD8, CD45RO, and CCR7 antigens and analyzed by flow cytometry. CD45RO and CCR7 expression was measured on CD4- or CD8-gated cells. Box plots depict the percentages of naive (CD45RO-CCR7+), central memory (CM, CD45RO+CCR7+), effector memory (EM, CD45RO+CCR7-), and effector memory RA (EMRA, CD45RO-CCR7-) cells in the CD4 **(A)** and the CD8 **(B)** subsets. Results are presented as in **Figure 1A**. ns, not significant.

The maturation status of CD4+ and CD8+ T cell was then assessed by analyzing the expression of markers specific for naïve (CD45RO-CCR7+), central memory (CM, CD45RO+CCR7+), effector memory (EM, CD45RO+CCR7-), and effector memory RA (EMRA, CD45RO-CCR7-) subsets. In both groups of patients, the CD4+ EM (**Figure 3A**) and the CD8+ EM and EMRA (**Figure 3B**) subsets were predominantly represented. However, no statistically significant differences were observed in the proportions of these subsets between Group 1 and Group 2 in either the CD4+ or the CD8+ compartments (**Figures 3A, B**).

**Figure 4:**
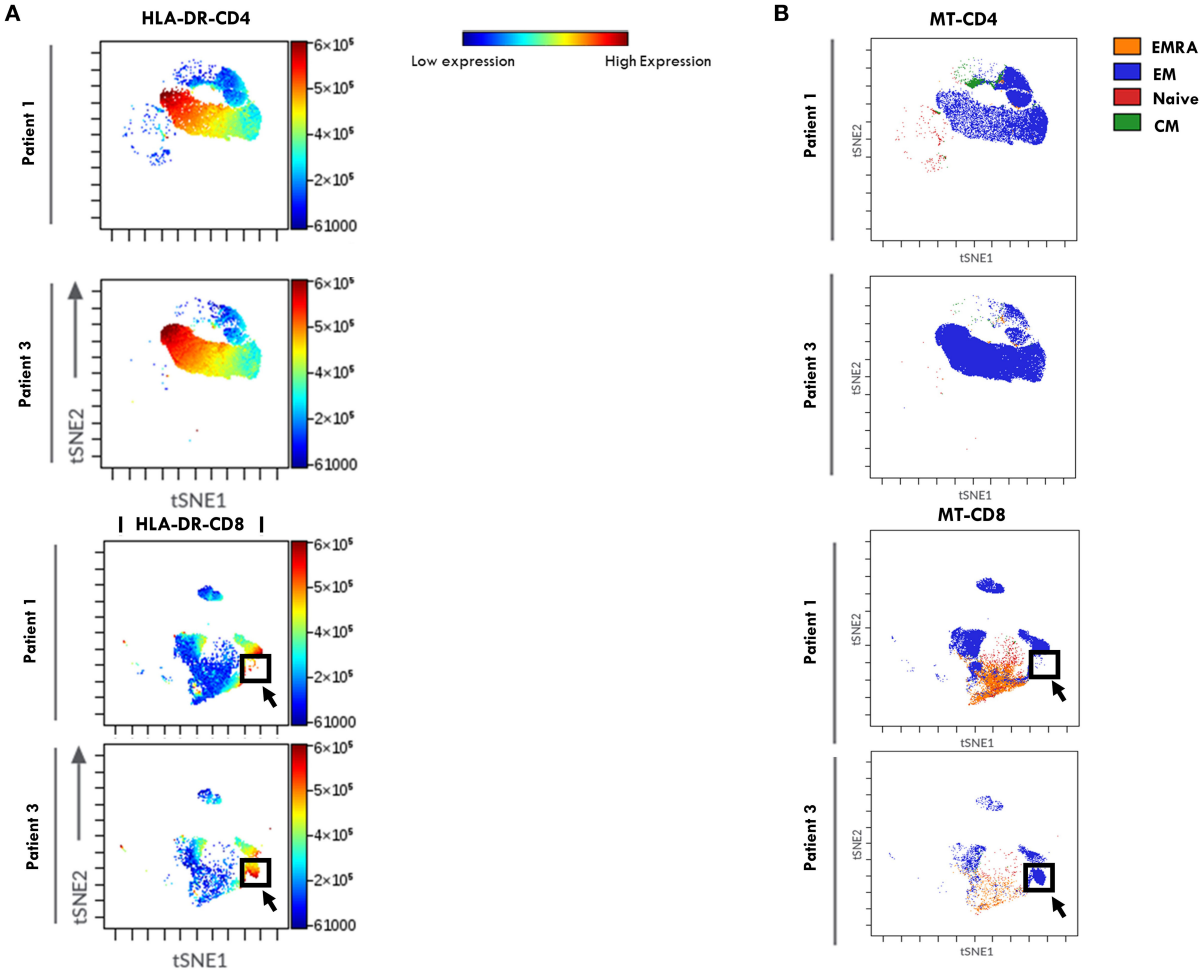
viSNE of HLA-DR expression in T Cell maturation subsets from SF of patients undergoing different disease courses. SFMCs from new-onset Oligoarthritis patients stained with Abs against CD3, CD4, CD8, CD45RO, CD45RA, CCR7, and HLA-DR markers were mapped using viSNE. Results are visualized as scatter plots, where each point represents a single cell in high-dimensional space and clusters of cells sharing similar phenotypes are grouped in defined regions of the plots. Cells are colored by marker expression levels. The axes are in arbitrary units. **(A)** viSNE plots show the distribution of the HLA-DR marker on CD4+ and CD8+ cell subsets in one representative patient from Group 1 (Patient 1) and Group 2 (Patient 3). The color scale represents the expression intensity of HLA-DR, with blue indicating low and red indicating high expression, enabling the comparison of expression patterns between the two patients. **(B)** FlowSOM quantitative clustering analysis on viSNE of the main T cell maturation subsets visualized using different colors: Naïve (red), EM (blue), EMRA (orange), and CM (green).

Dimensionality reduction analysis using viSNE, followed by FlowSOM clustering analysis, was then performed on the T cell flow cytometry data (**Figure 4**) to explore HLA-DR distribution pattern within T cell maturation subsets and to visualize potential group-specific differences between the two groups of patients. In the viSNE maps (**Figure 4A**), CD4^+^ and CD8^+^ cells formed distinct clusters, and HLA-DR expression levels, represented by a color scale, varied across the subsets. Notably, higher HLA-DR expression (indicated by a dark red signal) was observed within the CD4^+^ compartment of Group 2 patients compared to Group 1, consistent with the increase in activated CD4^+^ T cells reported in **Figure 2C**. FlowSom clustering revealed that the subset with the highest HLA-DR expression corresponded to the EM population (**Figure 4B**). While overall HLA-DR distribution profile in CD4+ cells was similar between groups, a distinct pattern was observed within the CD8 compartment. Specifically, Group 2 showed a unique cluster of HLA-DR^+^ CD8^+^ EM cells not observed in Group 1 (highlighted by a black arrow and box), indicating a differential activation profile associated with disease progression.

These data suggest that a high CD3:CD14 ratio combined with elevated percentages of HLA-DR+ CD4+ T cells in the SF of Oligoarthritis patients holds prognostic value for identifying, at disease onset, those at increased risk of developing polyarticular extension within two years of diagnosis, representing promising early immune biomarkers of disease progression.

### T cell activation state and maturation subset proportions in PB at onset discriminate between oligoarthritis patients likely to develop a persistent oligoarticular vs an extended polyarticular course

**Figure 5:**
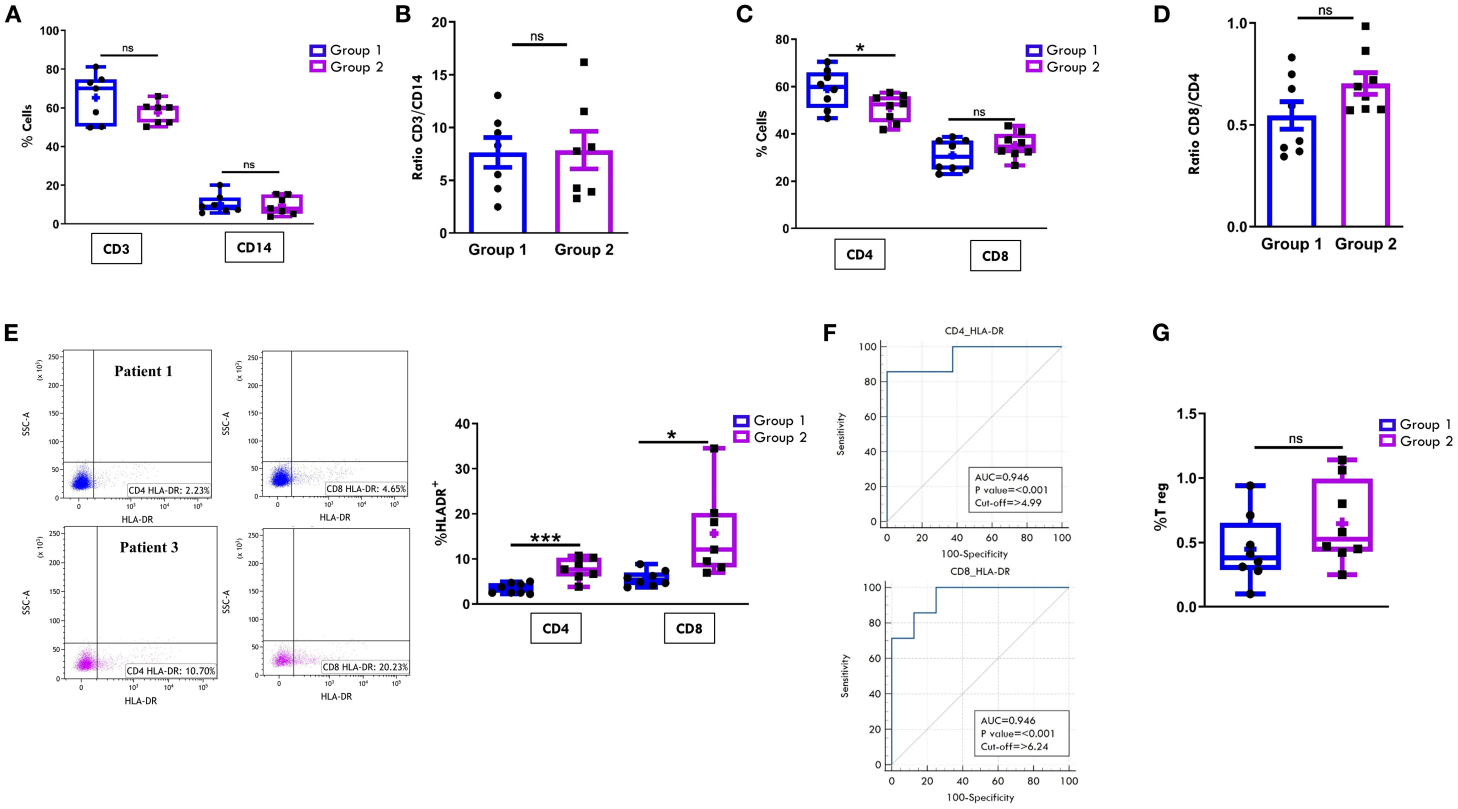
Comparative analysis of T cell and monocyte/macrophage populations in PB between patients undergoing different disease courses. PBMCs were purified from new-onset Oligoarthritis patients, stained with Abs against CD3, CD14, CD4, CD8, HLA-DR, CD25 and CD127 antigens, and analyzed by flow cytometry. **(A)** Box plots depict the median percentages of CD3+ and CD14+ cells in the two groups of patients. Results are presented as in **Figure 1A**. ns, not significant. **(B)** CD3:CD14 ratio in patients from Groups 1 and 2 is presented as a bar graph as in **Figure 1B**. ns: not significant. **(C)** Box plots depict the median percentages of CD4+ and CD8+ cells in patients from Groups 1 and 2. Results are presented as in **Figure 1A**. *P* values for CD4 cell percentages in Group 1 relative to Group 2: *p ≤ 0.05; ns: not significant. **(D)** CD8:CD4 ratio in patients from Groups 1 and 2 is presented as in **Figure 1B**. ns, not significant. **(E)** Flow cytometry plots (*left*) show HLA-DR positivity in one representative patient from Group 1 (patient 1) and one from Group 2 (patient 3). Results are presented as in **Figure 1A**. Box plots (*right*) show the median percentages of HLA-DR+ cells in CD4+ and CD8+ cell subsets. Results are presented as described in **Figure 1A**. *P* values for HLA-DR+CD4+ cell percentages in Group 1 relative to Group 2: ***p < 0.001; *p* values for HLA-DR+CD8+cell percentages in Group 1 relative to Group 2: *p ≤ 0.05. **(F)** ROC curves show the power of HLA-DR+CD4+ and HLA-DR+CD8+ cell percentages in discriminating between Group 1 and Group 2. Results are presented as in **Figure 1C**. *P* values for CD4+HLA-DR+ cell percentage in Group 1 relative to Group 2: p ≤ 0.001; *p* values for CD8+HLA-DR+ cell percentage in Group 1 relative to Group 2: p ≤ 0.001. **(G)** Box plots show the median percentages of T Reg+ cells from tested patients of Group 1 and 2. Results are presented as in **Figure 1A**. ns, not significant.

Comparison of T lymphocyte and MM composition in PBMCs at onset confirmed the expected higher proportions of CD3+ than CD14+ cells in both groups of patients (**Figure 5A**). Unlike observations in SF samples, no significant differences were found in the CD3:CD14 ratio between the two groups (**Figure 5B**).

The typical cytotoxic and helper T cell distribution in PB, with CD4+ cells approximately twice as abundant as CD8+ cells, was also maintained in both groups at onset. However, similarly to what observed in SFMCs, Group 2 patients showed a significantly lower proportion of CD4+ cells (50.87% ± 2.0 vs. 59.01% ± 2.9; p= 0.03) and a higher, though not statistically significant, percentage of CD8+ cells (35.24% ± 1.9 vs. 30.9% ± 2.3) compared to Group 1 (**Figure 5C**). As a result, the CD8:CD4 ratio was higher in Group 2 than in Group 1 (0.7 ± 0.05 vs. 0.5 ± 0.06), although this difference did not reach statistical significance (**Figure 5D**). Notably, analysis of HLA-DR expression revealed a significantly increased proportion of activated cells within both CD4+ (7.7% ± 0.9 vs 3.4% ± 0.4; p=0.0006) and CD8+ (15.6% ± 3.6 vs 5.6% ± 0.6; p= 0.013) subsets in Group 2 compared to Group 1 (**Figure 5E**), suggesting an association between elevated HLA-DR+ T cells in PB at onset and progression to a polyarticular disease course. These findings were further supported by ROC analysis (**Figure 5F**), which confirmed the high discriminating potential of this activation marker (CD4-HLA-DR AUC: 0.94, p<0.001; CD8-HLA-DR AUC: 0.94, p<0.001). Cut-off values associated with disease extension were identified as 4.9% for CD4^+^HLA-DR^+^ cells (sensitivity 85.71%; specificity 100%) and 6.2% for CD8^+^HLA-DR^+^ cells (sensitivity 100%; specificity 75%). Consistent with SF data, the frequency of Tregs in PB did not significantly differ between the two groups (**Figure 5G**).

**Figure 6:**
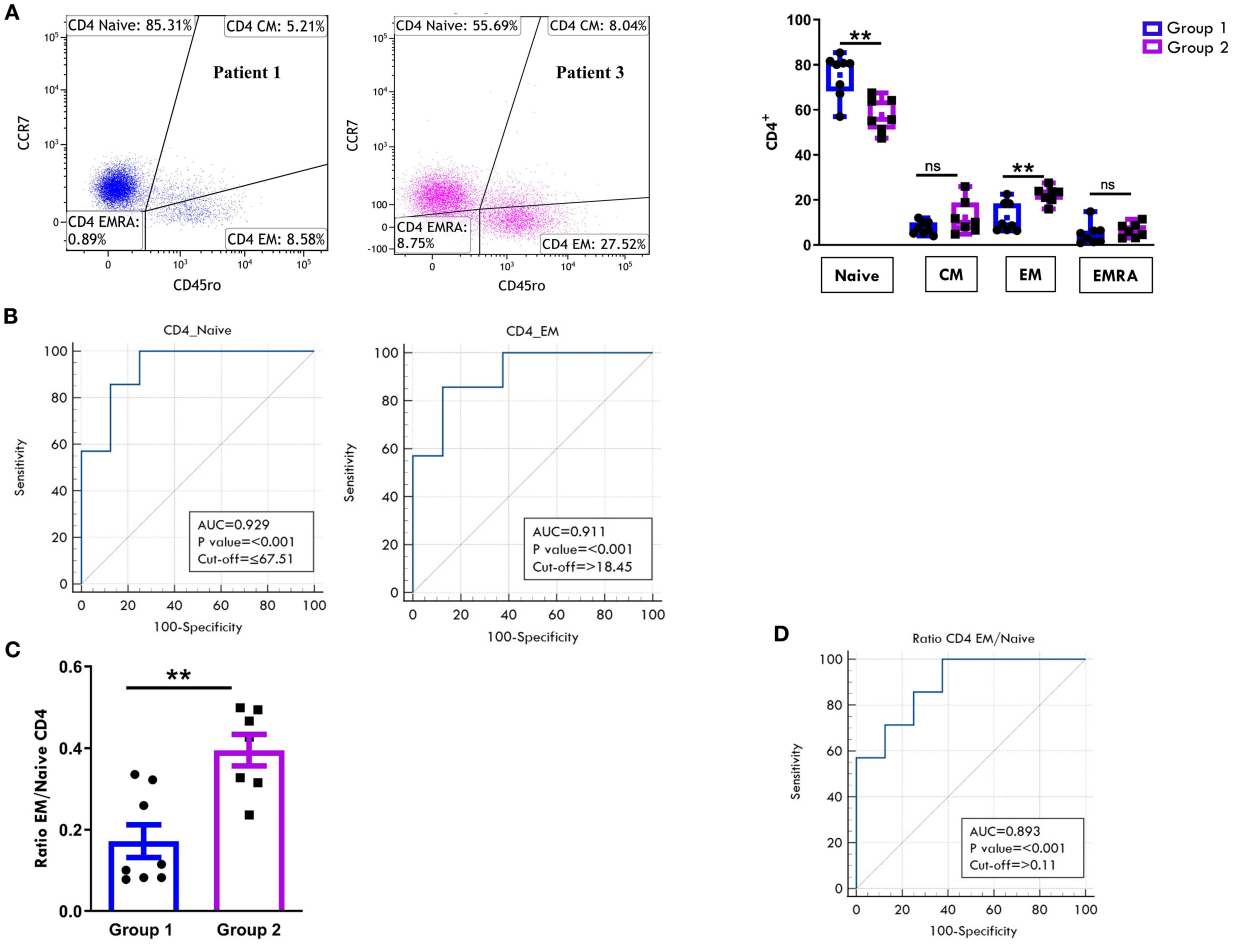
Comparative analysis of CD4 cell maturation subsets in PB between patients undergoing different disease courses. PBMCs were stained with Abs against CD4, CD45RO, and CCR7 antigens and analyzed by flow cytometry. CD45RO and CCR7 expression was measured on CD4-gated cells. **(A)** Flow cytometry plots (*left*) show maturation marker expression on one representative patient from Group 1 (patient 1) and one from Group 2 (patient 3). Results are presented as described in **Figure 1A**. Box plots (*right*) showing subset percentages are presented as in **Figure 1A**. *P* values for Naive CD4+ cell percentage in Group 1 relative to Group 2: **p ≤ 0.01; *P* values for EM CD4+ percentage in Group 1 relative to Group 2: **p ≤ 0.01; ns: not significant. **(B)** ROC curves show the prognostic value of Naive and EM cell percentages in the CD4 subset. Results are presented as in **Figure 1C**. *P* values for Naive CD4+ cell percentage in Group 1 relative to Group 2: p ≤ 0.001; *P* values for EM CD4+ cell percentage in Group 1 relative to Group 2: p ≤ 0.001. **(C)** EM: Naive ratio in the two groups of patients is presented as a bar graph as in **Figure 1B**. *P* values for EM: Naive CD4+ ratio in Group 1 relative to Group 2: **p ≤ 0.01. **(D)** ROC curve shows the discriminating value of the EM: Naive ratio. Results are presented as in **Figure 1C**. *P* values for the EM: Naive ratio in Group 1 relative to Group 2: p ≤ 0.001.

**Figure 7:**
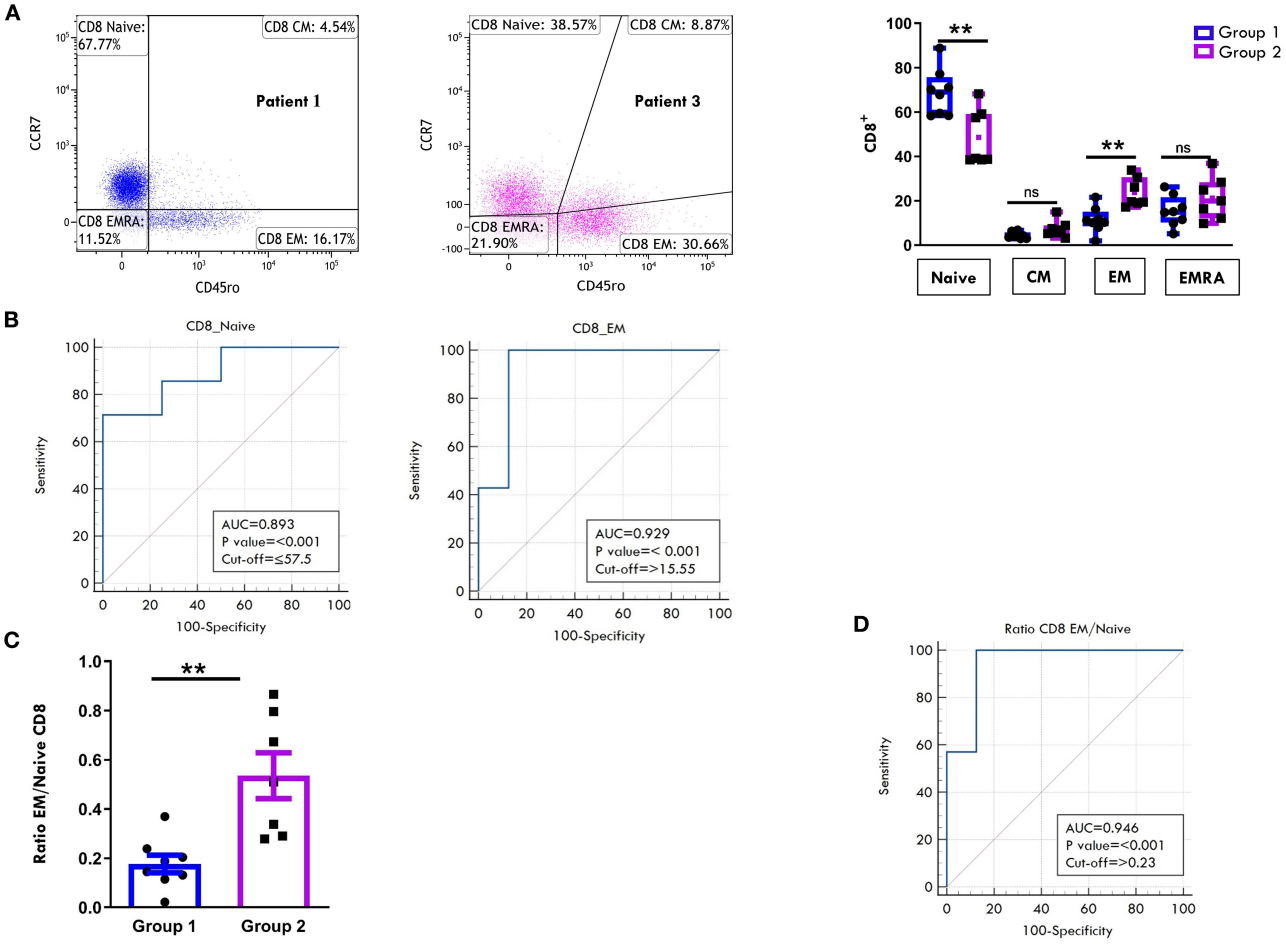
Comparative analysis of CD8 cell maturation subsets in PB between patients undergoing different disease courses. PBMCs were stained with Abs against CD8, CD45RO, and CCR7 antigens and analyzed by flow cytometry. CD45RO and CCR7 expression was measured on CD8-gated cells. **(A)** Flow cytometry plots (*left*) show results of maturation marker positivity of one representative patient from Group 1 (patient 1) and one from Group 2 (patient 3). Results are presented as described in **Figure 1A**. Box plots (*right*) display subset percentages as presented in **Figure 1A**. *P* values for Naive CD8+ cell percentages in Group 1 relative to Group 2: **p ≤ 0.01; *P* values for EM CD8+ cell percentage in Group 1 relative to Group 2: **p ≤ 0.01; ns, not significant. **(B)** ROC curves show the discriminating value of Naive and EM cell percentages in the CD8 subset. Results are presented as in **Figure 1C**. *P* values for Naive CD8+ cell percentages in Group 1 relative to Group 2: p ≤ 0.001; *P* values for EM CD8+cell percentages in Group 1 relative to Group 2: p ≤ 0.001. **(C)** EM: Naive ratio in the two groups of patients is presented as in **Figure 1B**. *P* values for the EM: Naive CD8+ ratio in Group 1 relative to Group 2: **p ≤ 0.01. **(D)** ROC curves show the discriminating value of the EM : Naive CD8 ratio. Results are presented as in **Figure 1C**. *P* values for the EM: Naive CD8+ ratio in Group 1 relative to Group 2: p ≤ 0.001.

Regarding the T cell maturation state, we observed a predominance of naïve over memory T cell subsets in both the CD4+ (**Figure 6A**) and CD8+ (**Figure 7A**) compartments at onset, regardless of clinical outcome. However, patients in Group 1 exhibited significantly higher proportions of naïve CD4+ (75.4% ± 3.3 vs 57.8% ± 2.8; p=0.0017) and CD8+ (68.9% ± 3.7 vs 48.5% ± 4.7, p=0.0048) cells compared to Group 2. Conversely, EM T cells were significantly more abundant in Group 2, both within the CD4+ (22.2% ± 1.3 vs 12.1% ± 2.3; p=0.003) and CD8+ (23.6% ± 2.4 vs 11.3% ± 2.0, p=0.0019) populations (**Figures 6A**, **7A**). ROC curves identified optimal cut-off values for distinguishing between disease courses. For naïve T cells, thresholds of 67.5% for CD4+ (sensitivity: 100%, specificity: 75%) and 57.5% for CD8^+^ (sensitivity: 71.43%, specificity: 100%) were established, with high AUC values and strong statistical significance (CD4^+^ AUC: 0.92, *p* < 0.001; CD8^+^ AUC: 0.89, *p* < 0.001). For EM subsets, cut-offs were 18.4% for CD4^+^ (sensitivity: 85.71%, specificity: 87.5%) and 15.5% for CD8^+^ cells (sensitivity: 100%, specificity: 87.5%), also showing excellent performance (CD4^+^ AUC: 0.91, *p* < 0.001; CD8^+^ AUC: 0.92, *p* < 0.001) (**Figures 6B**, **7B**). As depicted in **Figures 6C, D**, **7C, D**, the EM:naïve cell subset ratio was significantly increased in Group 2 compared to Group 1 in both CD4+ (0.3 ± 0.03 vs ratio 0.1 ± 0.04 vs, p= 0.0016) and CD8+ (0.5 ± 0.09 vs 0.17 ± 0.03 vs, p=0.002) populations. This ratio, thus, emerged as a particularly effective marker for disease progression. ROC analysis confirmed its prognostic value, with optimal cut-off values of 0.11 for CD4^+^ cells (AUC: 0.893; *p* < 0.001; sensitivity: 100%, specificity: 62.5%) and 0.23 for CD8^+^ cells (AUC: 0.946; *p* < 0.001; sensitivity: 100%, specificity: 87.5%).

**Figure 8:**
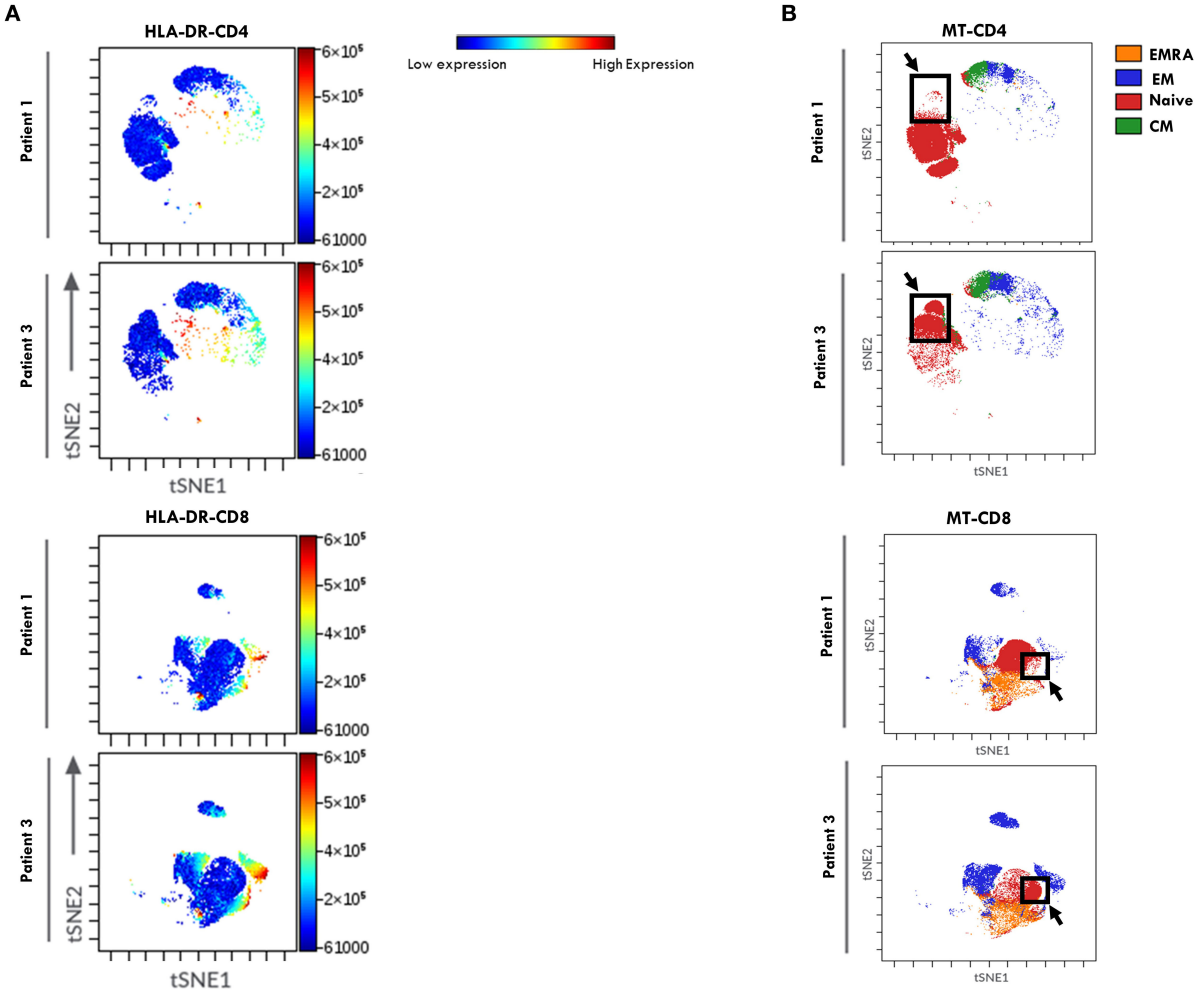
viSNE of HLA-DR expression in T Cell maturation subsets from PB of oligoarthritis patients undergoing different disease courses. PBMCs stained with Abs directed to CD3, CD4, CD8, CD45RO, CD45RA, CCR7, and HLA-DR markers were analyzed by viSNE. Results are presented as in **Figure 4**.

ViSNE dimensionality reduction analysis of PB T cell FACS data confirmed higher HLA-DR expression levels in both CD4+ and CD8+ cell compartments in patients from Group 2, consistent with findings reported in **Figures 5E**. However, no substantial differences in HLA-DR^+^ cell distribution patterns were observed between the two groups of patients (**Figure 8A**). FlowSom clustering further validated the increased proportion of EM T cells and the reduced proportion of naive T cells in both the CD4+ and CD8+ subsets in Group 2 compared to Group 1. Additionally, this analysis revealed distinct patterns of naïve T cell distribution between the two groups (as indicated by the arrows, **Figure 8B**). As observed in SF, the highest HLA-DR expression intensity (shown in red, **Figure 8A**) was localized predominantly in EM T cells.

Taken together, our data indicate that specific T cell parameters measured in the PB of Oligoarthritis patients at disease onset, such as the frequency of HLA-DR+ cells, the proportions of naive and EM subsets, and the EM: Naïve ratio in both CD4+ and CD8+ compartments, may hold strong prognostic value, potentially identifying patients at increased risk of developing polyarticular extension within 2 years from diagnosis, representing promising early biomarkers of disease progression.

### Assessment of CD14^+^ cell polarization state reveals no prognostic value in oligoarthritis progression

The polarization states of the MM populations in SFMCs and PBMCs at disease onset were then assessed by analyzing the expression of CD80 (M1 marker) and CD206 and CD163 (M2 markers) within CD14+ gated cells (**Supplementary Figure S2**). We also evaluated the presence of mixed phenotypes characterized by coexpression of CD80 with CD206 or CD163. In SF, a higher proportion of CD14+ cells expressed the CD163 marker, accounting for ≈45% of the gated population, compared to ≈35% cells expressing either CD80 or CD206 across both patient groups (**Supplementary Figure S2A**). Only about 20% of CD163+ cells coexpressed CD206, indicating an incomplete polarization toward the M2 phenotype. Mixed M1/M2 subsets were also detected, with ≈21% of CD163+ and ≈14% of CD206+ cells co-expressing CD80. No statistically significant differences in the distribution of polarization subsets were found between patients who would undergo different clinical courses at follow up (**Supplementary Figure S2A**).

In PB, the analysis of CD14+-gated cells revealed that the CD163+ M2 subset was the most prevalent (≈41%) in both patient groups, indicating a general skewing toward the M2 polarization. However, expression of CD206 was minimal (~3%), whether alone or in combination with CD163, again suggesting an incomplete M2 polarization. While Group 2 showed higher, tough not statistically significant, percentages of cells expressing the M1 marker CD80 (15.5% ± 7.3 vs. 9.5% ± 3.5) as well as of cells exhibiting a mixed M1/M2 (CD80^+^/CD163^+^) phenotype (13.4% ± 6.6 vs. 4.7% ± 1.3) compared to Group 1, the proportion of all other polarization subsets did not differ between groups (**Supplementary Figure S2B**).

These findings indicate that the proportions of M1 and M2 subsets in both SF and PB are comparable at disease onset, regardless of subsequent clinical course, suggesting that MM polarization status at diagnosis cannot represent a biomarker of disease extension in Oligoarthritis.

### TREM-1 surface expression on CD14+ cells and sTREM-1 levels in PB and SF at onset distinguish oligoarthritis patients with different clinical courses

**Figure 9:**
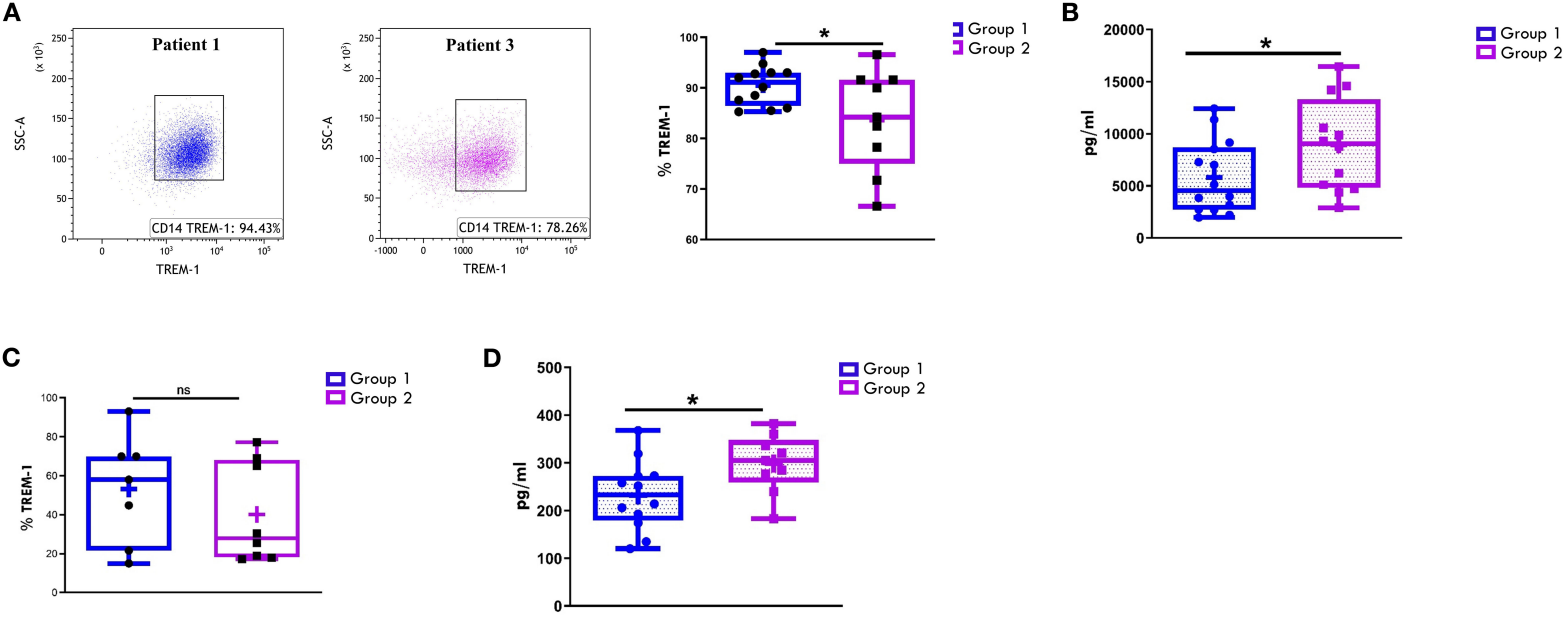
Comparative analysis of TREM-1 expression on CD14+ cells and sTREM-1 release in patients undergoing different disease courses. SFMCs **(A)** and PBMCs **(C)** were stained with Abs against CD14 and TREM-1 antigens and analyzed by flow cytometry. sTREM-1 release in SF **(B)** and PB **(D)** was quantified by ELLA. **(A)** Flow cytometry plots (*left*) show TREM-1 expression on CD14+ cells from SFMCs in one representative patient from Group 1 (patient 1) and one from Group 2 (patient 3). **(A, C)** Box plots (*right*) showing TREM-1+ cell median percentages are presented as in **Figure 1A**. *P* values for TREM-1 percentages in Group 1 relative to Group 2: *p ≤ 0.05; ns, not significant. **(B, D)** sTREM1 secretion data are presented in pg/ml as a box plot. Individual data points represent each single patient. *P* values for sTREM-1 concentrations in Group 1 relative to Group 2: *p ≤ 0.05.

We have previously identified the immunoregulatory receptor, TREM1, as a novel hypoxia-inducible marker in monocytic lineage cells infiltrating the inflamed joints of children affected by Oligoartritis (61, 62, 69, 70). To evaluate TREM1 potential as a marker of disease extension, we compared its expression in the two groups of patients by measuring both the levels of the transmembrane glycoprotein on CD14+- gated cells and of its soluble form (sTREM1) in SF and PL samples. As shown in **Figure 9A**, the majority of CD14+ cells in SF from both groups at diagnosis expressed membrane-bound TREM1. However, Group 1 patients showed a significantly higher proportion of TREM-1^+^ CD14^+^ cells compared to Group 2 (90.47% ± 1.11 vs. 83.6% ± 3.3; *p* = 0.04) (**Figure 9A**). An inverse relationship was observed with sTREM-1 concentrations in SF, which were significantly higher in Group 2 *vs* Group 1 (8926 +/-1282 vs 5822+/-931.6; p value:0.05) (**Figure 9B**). In PB samples, both the percentage of TREM-1+ CD14+ cells and sTREM-1 levels were lower than in SF samples (**Figures 9C, D**), confirming previous reports (61). However, similarly to SF, PB from Group 2 patients exhibited a lower, though not statistically significant, percentage of TREM1+ cells (40.16 +/- 9.079 vs 53.21 +/-10.59) (**Figure 9C**) and significantly higher sTREM1 levels (298.8 +/-20.45 vs 232.0 +/21.01; p value: 0.03) compared to Group 1 (**Figure 9D**).

These findings suggest that reduced TREM1 expression levels on SFMCs- and PBMCs-derived CD14+ cells, combined with elevated sTREM-1 concentrations in SF and PB, at disease onset may serve as early biomarkers of polyarticular extension in Oligoarthritis patients.

### Surface marker expression profiling of SF-derived EVs identifies potential biomarkers of disease extension in oligoarthritis patients

**Figure 10:**
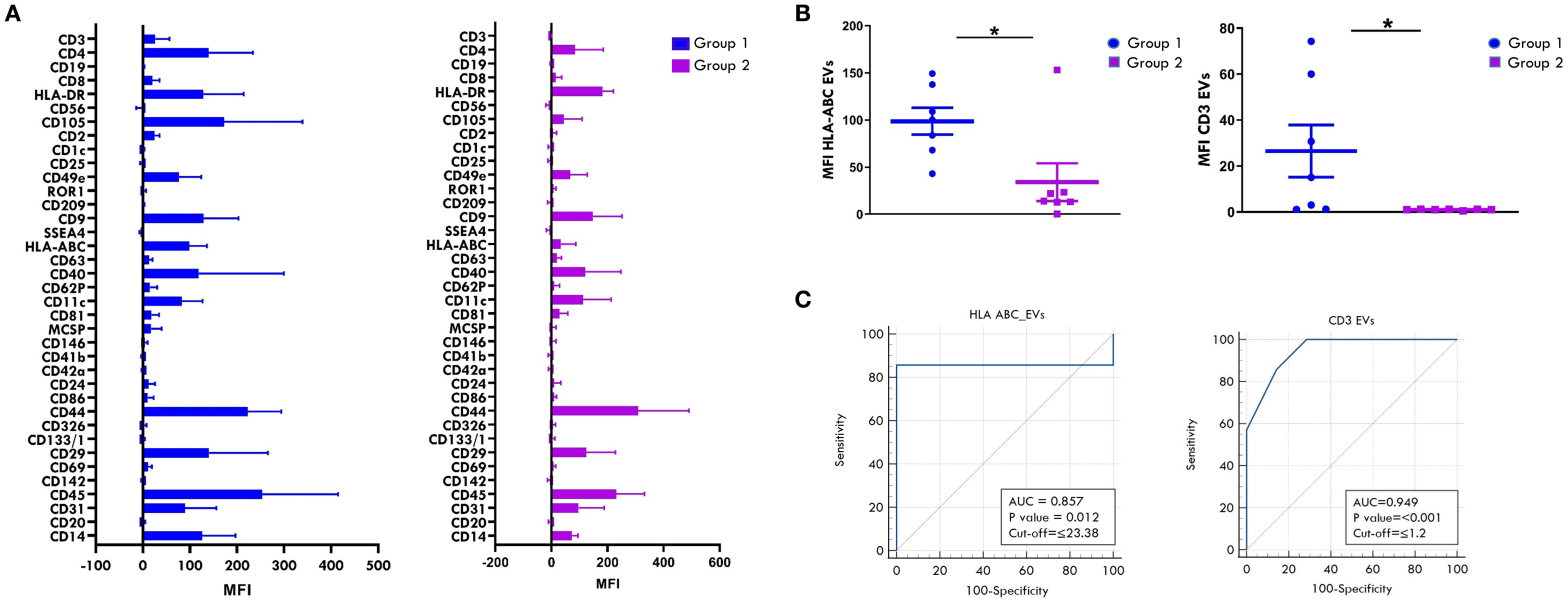
Comparative analysis of surface antigen profile on SF-derived EVs between patients undergoing different disease courses. Surface expression levels of 37 different antigens was evaluated by MACSPlex analysis on EVs released into SF from new-onset Oligoarthritis patients. **(A)** Bar graphs show the levels of 31 of the 37 tested surface molecules found expressed in patients from Group 1 and/or Group 2. Results represent the means ± SEM of the Median Fluorescence Intensity (MFI) values. **(B)** Scatter dot plot depicts the expression level of HLA-ABC and CD3 antigens in patients from Groups 1 and 2. Each dot represents one single patient. Horizontal line indicates the median MFI value for each group. *P* values for HLA-ABC and CD3 levels in patients from Group 1 relative to Group 2: *p ≤ 0.05. **(C)** ROC curves show the power of HLA-ABC and CD3 expression levels in discriminating between Group 1 and Group 2. Results are presented as in **Figure 1C**. *P* values for HLA-ABC expression levels in Group 1 relative to Group 2: p ≤ 0.01; *P* values for CD3 levels in Group 1 relative to Group 2: p ≤ 0.001.

Our previous studies demonstrated that EV proteomic signatures in biological fluids from Oligoarthritis patients may represent potential early biomarkers for patient stratification based on clinical outcome (55). In this study, we conducted a comparative analysis of the surface protein cargo of SF-derived EVs from a subset of 14 new-onset Oligoarthritis patients (n=7 in Group 1 and n= 7 in Group 2) to identify additional early biomarkers able to discriminate patients at higher risk of polyarticular extension. Patients included in the analysis were selected from our original proteomics cohort based on the availability of sufficient volumes of cell-free SF samples, to ensure consistency with previous analyses and minimize variability related to sample handling, processing, or patient characteristics. EVs were isolated and analyzed using a multiplex bead-based flow cytometry approach (65) with the MACSplex Exosome Kit (66). Expression patterns and levels of 37 surface antigens on EVs were simultaneously measured, as described in Refs (66, 71), and compared between the two clinical groups. As shown in **Figure 10A** and detailed in **Table 2**, patients undergoing different disease courses exhibited distinct surface protein profiles at onset. A total of thirty antigens were detected (MFI> 0) on SF-derived EVs from Group 1 patients, while twenty-seven on those from Group 2.

**Table 2:** Surface antigen expression profile in SF-derived EVs from new-onset Oligoarthritis patients.

Category	Epitopes	MFI	
		Group 1	Group 2
Immune system	CD2	25,18	6,08
	CD40	118,99	121,43
	CD14	126,41	70,21
	CD3	26,49	0,98
	CD4	140,23	84,58
	CD19	0,20	0,00
	CD8	20,45	16,33
	CD86	10,58	10,34
	CD45	253,57	231,80
	CD20	1,44	0,00
	CD1c	0,82	0,00
Endothelium	CD31	89,99	96,76
	CD105	172,79	44,81
	CD146	4,82	5,20
Extracellular vesicles	CD81	18,37	29,20
	CD9	129,03	148,35
	CD63	13,05	19,78
MHC-Associated	HLA-ABC	98,72	34,10
	HLA-DR	128,95	183,08
Integrins	CD62P	15,01	9,69
	CD69	11,42	7,55
	CD49e	76,68	67,22
	CD29	140,55	124,17
	CD11c	83,25	112,99
	CD44	223,32	309,44
Other	ROR1	2,93	7,34
	MCSP	17,61	4,31
	CD24	12,37	10,19
	CD326	1,60	5,50
	CD133/1	1,53	2,41

List of the 30 EV membrane antigens expressed in one or both clinical groups. Antigen expression levels are indicated as mean of median fluorescence Intensity (MFI).

Classical EV markers, such as the tetraspanins, CD9, CD81, and CD63, were expressed in both groups, with CD9 being the most abundant. Several immune-related antigens (CD2, CD40, CD14, CD3, CD4, CD8, CD86, CD45) were also detected across all patients, suggesting a likely origin from immune cells infiltrating the inflamed joint. Among them, CD45, CD40, CD4, and CD14 were the most prominently expressed in both groups. Only three immune-related antigens, CD19, CD20, and CD1c, were exclusively detected on EVs from Group 1 patients, albeit at low levels, with CD20 being the most represented. The endothelial markers, CD31 and CD105, were highly expressed in both groups, supporting a derivation from endothelial-derived EVs. Furthermore, various integrins (CD29, CD11c, CD49e, CD44) and MHC-associated molecules (HLA-ABC and HLA-DR) were detected across both groups.

Comparison of commonly expressed surface antigens revealed significantly higher levels of HLA-ABC and CD3 antigens on SF-EVs from Group 1 compared to Group 2 (HLA-ABC: 98.72 ± 14.2 vs 34.1 ± 20, p value: 0.02; CD3: 26.49 ± 11.34 vs 0.98 ± 0.08, p value: 0.04) (**Figure 10B**). ROC curve analyses showed high discriminative power for both markers, with AUC of 0.857 for HLA-ABC (*p* = 0.012) and 0.949 for CD3 (*p* < 0.001). Optimal cut-off values indicative of polyarticular extension were 23.38 for HLA-ABC (sensitivity 85.7%, specificity 100%) and 1.2 for CD3 (sensitivity 100%, specificity 71.4%) (**Figure 10C**). These findings suggest that HLA-ABC and CD3 expression levels on SF-EVs at disease onset may represent putative early biomarkers of polyarticular progression in new-onset Oligoarthritis patients, demonstrating good sensitivity and specificity.

### Combination of immunological markers and clinical features for early risk stratification

**Figure 11:**
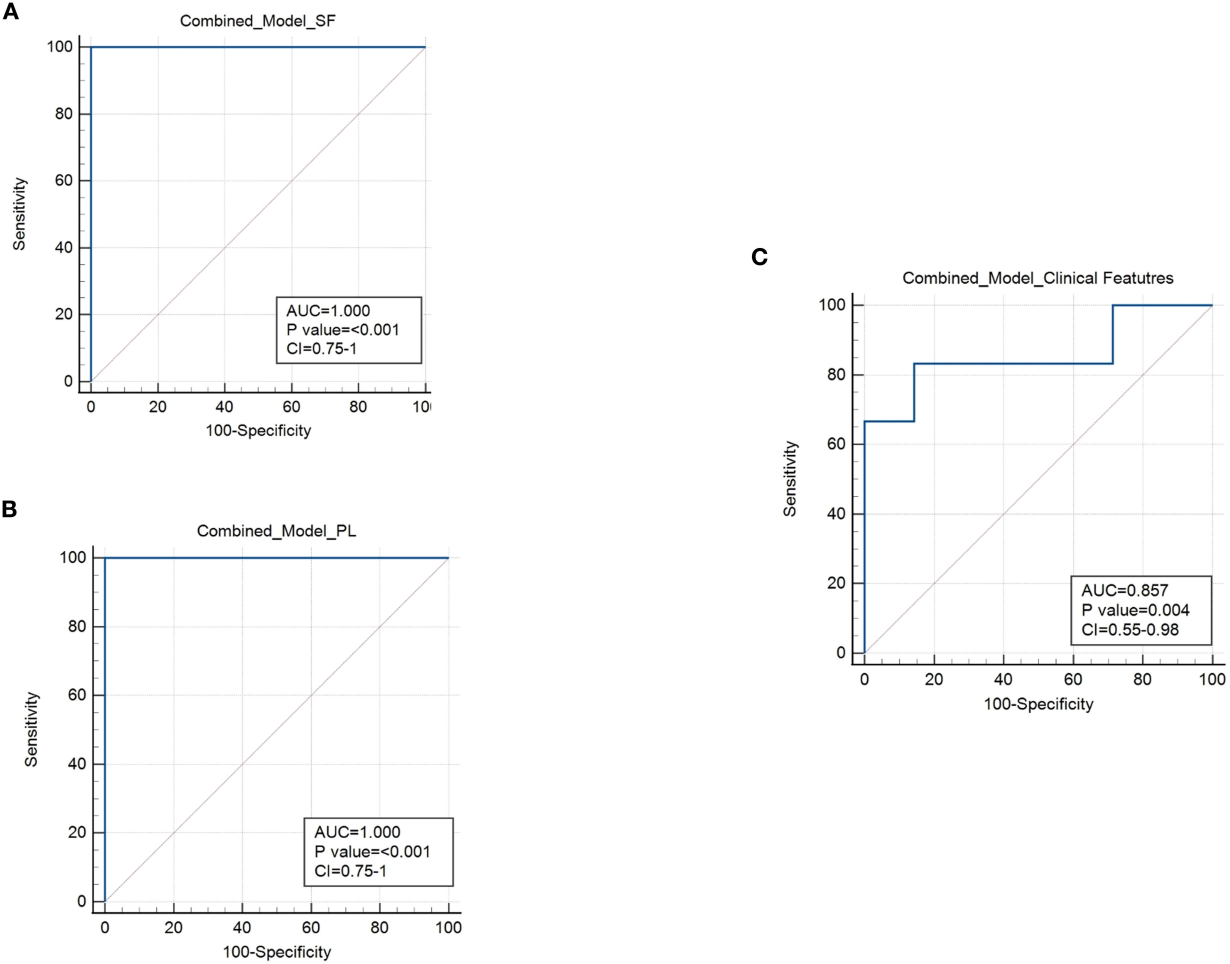
Combined ROC analysis of prognostic markers for the early stratification of oligoarthritis patients at risk of extension. **(A)** ROC curve shows the discriminating power of the combination of CD3:CD14 ratio and EV HLA-ABC and CD3 expression biomarkers in SF. Results are presented as in **Figure 1C**. AUC, p values, and Confidence Interval (CI) are reported for each graph. P values of Group 1 relative to Group 2: p ≤ 0.001. **(B)** ROC curve shows the discriminating value of the combination of HLA-DR+ CD4+ and CD8+ T cell percentages, naive and EM cell proportions and EM:naive ratio in CD4+ and CD8+ subsets biomarkers in PB. Results are presented as in **Figure 1C**. AUC, p values, and Confidence Interval (CI) are reported for each graph. P values of Group 1 relative to Group 2: p ≤ 0.001. **(C)** ROC curve shows the discriminating power of the combination of ESR levels, number of active joints, and number of patients with symmetric joint presentation. Results are presented as in **Figure 1C**. AUC, p values, and CI are reported for each graph. P values of Group 1 relative to Group 2: p = 0.004.

The prognostic accuracy of biomarker-based approaches can be significantly enhanced by combining multiple markers (67, 72–74). We, therefore, assessed whether the discriminatory power of the immunological prognostic biomarkers identified in SF or PB could be improved when used in combination. For SF we considered as biomarkers the CD3:CD14 cell ratio and the expression levels of HLA-ABC and CD3 on EVs, whereas for PB selected markers included the percentage of HLA-DR T cells, proportions of naive and EM subsets, and the EM:naive ratio within both CD4+ and CD8+ T cell compartments. To evaluate the combined discriminative ability of these markers, we applied generalized linear modeling (GLMNET) with a LOOCV approach. The results demonstrated that the immunomarker combinations significantly outperformed individual biomarkers, achieving optimal prognostic performance for both SF (**Figure 11A**) and PB (**Figure 11B**), with AUC of 1.0, p value< 0.001, and CI ranging between 0.75 to 1 (sensitivity 100%; specificity 100%). Next, we applied GLMNET analysis to the clinical variables that were significantly different between Group 1 and Group 2 at onset, namely ESR levels, number of active joints, and symmetric joint involvement (**Table 1**). This model also showed good discriminatory power (AUC: 0.857; *p* = 0.004; CI: 0.55–0.98; sensitivity: 83.3%; specificity: 85.7%) (**Figure 11C**). However, the classification performance of the clinical marker combination was lower than that of the immunological biomarker panels in terms of both AUC and statistical significance.

These findings suggest that the combinations of immunomarkers from SF and PB may offer improved stratification of the analyzed cohort of Oligoarthritis patients at disease onset in relation to their risk of polyarticular extension within two years of follow-up, compared to clinical parameters, and could thus potentially represent clinically relevant tools for early risk assessment, pending independent validation in larger cohorts.

## Discussion

The identification of low-invasive biomarkers for the early stratification of Oligoarthritis patients remains a critical unmet need. Currently, no clinical or laboratory biomarkers are routinely used in clinical practice to reliably predict disease extension at diagnosis. This is the first study to comprehensively characterizes T cell and MM subset composition, activation, and maturation states in both SF and PB from treatment-naïve Oligoarthritis patients at disease onset, along with surface protein expression pattern of SF-derived EVs, to prospectively identify early immune biomarkers indicative of subsequent polyarticular extension. Our findings reveal distinct immune cell and EV profiles associated with different disease courses over a two-year follow-up, offering novel candidate biomarkers for early prognosis and providing mechanistic insights into disease progression that may support more personalized therapeutic strategies.

Clinical differences among Oligoarthritis patients with divergent disease courses have been previously reported (9, 10, 21, 24, 75, 76). The clinical characteristics of our cohort are consistent with and reinforce these earlier findings, showing that patients who would progress to polyarthritis (Group 2) had a higher number of active joints, more frequent symmetric and/or upper limb involvement, ankle disease, and elevation of inflammatory markers at onset compared to those maintaining a persistent oligoarticular course (Group 1). Among them, ESR levels, active joint count, and symmetric joint presentation were significantly different between groups, discriminating patients at higher risk of disease extension. Interestingly, similar variables, have been previously shown to predict failure to achieve inactive disease in JIA patients after 18 months (73) or the risk of polyarticular extension in children with Oligoarthritis over long-term follow-ups (five years or more) (23, 74, 77). A significant increase in upper limb and symmetric joint involvement, along with elevated disease activity markers (e.g. ESR), have also been described in patients with extended JIA within the first two years (76), and clinical trials identified high ESR levels as a risk factor for arthritis flare (13). Although specific joint patterns, such as wrists, ankles, or small hand/foot joints, have been proposed as early predictors of polyarticular extension (23, 24, 75), we observed only a trend toward increased ankle and finger joint involvement in Group 2, which did not reach statistical significance. In addition, in contrast to Al-Matar et al. (23), we did not observe a significant association between polyarticular progression and ANA positivity, possibly due to differences in study design (prospective vs retrospective) or follow-up duration (2 years vs 5 years). Likewise, contrary to the findings by Habib et al. (76), we found no significant differences in age and CRP levels, which may reflect differences in the timing of evaluation (baseline *vs* first two years). In line with evidence indicating that combining clinical parameters improves prognostic accuracy of biomarker-based approaches (73, 74, 78), we observed that a composite score based on the three clinical parameters that significantly differed between the two groups, namely ESR, active joint count, and symmetric joint involvement, showed reasonable discriminative ability (AUC of 0.857) in ROC analysis. However, this combined clinical panel remains suboptimal, highlighting the need for additional biological biomarkers to enhance early risk stratification, particularly in patients who do not yet exhibit overt clinical signs of disease extension.

Among the immune biomarkers identified through SFMCs cytofluorimetric analysis at disease onset, the CD3:CD14 ratio appeared to have a good prognostic relevance. Specifically, using a threshold of 3.97, it could be associated with polyarticular extension within two years with an estimated 83% probability showing statistical significance, comparable to the discriminative ability of the combined clinical feature panel. As opposed to our findings, Hunter et al. (37) reported similar percentages of CD3+ and CD14+ cells in the SF of children with persistent and extended-to-be disease courses. Differences observed between these findings may possibly due to the different follow-up duration of the studies (1 year vs 2 years). Our results suggest a more pronounced T cell-mediated response at onset in the joints of patients who would develop polyarticular progression and a MM-driven proinflammatory response in those who would maintain an oligoarticular course. Notably, no significant differences in the CD3:CD14 ratio were observed in PB, indicating that this marker may be specifically relevant at the inflammatory site.

CD8+ T cells have been implicated in Oligoarthritis pathogenesis (7). In the present study, both patient groups showed a higher overall proportion of CD8+ than CD4+ T cells in SF of at diagnosis, but a significantly increase in the CD8:CD4 ratio was associated with a greater likelihood of disease progression to a polyarticular course within two years. These findings support and extend the trend reported by Hunter et al. (37), who also found a higher CD8:CD4 ratio in patients whose disease would extend at one year of follow-up. Conversely, other studies reported higher CD4:CD8 ratios in the joints of Oligoarthritis patients who had already progressed to an extended disease phenotype (7, 79). Taken together, these results suggest that CD8+ cell enrichment in inflamed joints may occur during the initial phases of the disease, potentially representing an early regulatory mechanism of disease progression, while a shift towards increased CD4+ T cell infiltration may emerge as the disease becomes more chronic, indicating a dynamic evolution of immune responses over time with CD4^+^ T cells playing a more prominent role in later stages.

HLA-DR up-regulation is a known marker of T cell activation in RA and other inflammatory arthritides. HLA-DR-presented peptides have been identified not only in synovial tissue but also in SFMCs and PBMCs (80). In JIA, activated T cells were found enriched in the inflamed joints of children with Oligoarthritis positively correlating with markers of disease activity, such as ESR and CRP levels (7, 79), and circulating HLA-DR+ T cells have also been described (81, 82). Consistent with these findings, we observed significant enrichment of HLA-DR+ T cells in both SF and PB at diagnosis, with particularly higher proportions in SF CD4+ T cells from patients who progressed to extended disease at follow-up. Circulating HLA-DR+ T cell proportion within both CD4+ and CD8+ compartments were also significantly elevated in these patients demonstrating superior discriminative ability compared to the combined panel of clinical variables, as evidenced by ROC analysis (AUC: 0,946). These findings highlight HLA-DR+ T cells as a robust early biomarker of disease extension in Oligoarthritis, especially when measured in PB. From a clinical perspective, the identification of circulating immune markers is particularly valuable, due to their ease of accessibility and minimal invasive nature, suitable for broad patient screening even in cases where SF volumes are insufficient or aspiration is not feasible. It is worth noting that previous studies have reported contrasting results, with higher HLA-DR+ T cell frequencies observed in patients who had maintained the oligoarticular form rather than those who had already progressed to polyarthritis (7). These discrepancies may reflect differences in T cell activation between early and advanced disease stages. Elevated activation may characterize the initial phases in patients who will follow a more severe clinical course, supporting the notion that these cells play a key role in initiating or driving disease progression. As the disease evolves, T-cell activation may subsequently decline, potentially reflecting adaptation to chronic inflammatory stimuli or immunoregulatory feedback mechanisms. This temporal evolution of immune signatures as the disease transitions from early inflammatory events to more established chronic pathology underscores the importance of assessing T cell activation early in the disease course when it may offer the greatest prognostic insight.

Another key finding of this study is the identification of naïve and EM CD4+ and CD8+ T cell subset proportion and ratios in PB as candidate prognostic markers. Despite naïve T cells being the predominant subset in both outcome groups, a significantly higher proportion was observed in patients who would maintain an oligoarticular course. In contrast, patients who later developed disease extension exhibited a significantly increased proportion of EM T cells and a significantly higher EM:naïve cell ratio. Importantly, these differences demonstrated strong prognostic performance, with ROC analysis yielding AUC values ranging from 0.89 to 0,946, exceeding those of combined clinical variables. These findings suggest that the distribution of circulating naïve T cells may represent a valuable tool for early patient stratification. It is worth noting that no significant differences in T cell maturation subsets were observed in SF, suggesting that these markers may hold specific systemic rather than local prognostic relevance. However, within the CD8+EM T cell compartment in SF, a distinct HLA-DR high population was consistently detected in the SF of Group 2 patients but absent in Group 1. This phenotype, indicative of chronic antigen exposure and sustained local T cell activation, may represent a joint-specific immune marker of patients at higher risk of polyarticular progression. Although proliferation markers such as Ki-67 were not included in the current panel due to limited cell numbers, the highly activated phenotype of this subset suggests potential local expansion. This possibility is supported by previous findings in RA, where synovial EM CD8^+^ T cells have been shown to express activation and proliferation markers such as CD80, CD86, PD-1, and Ki-67 (83) Future studies incorporating broader phenotypic and functional analyses, including the assessment of proliferation markers, will be essential to define the role of this population in disease progression and assess its prognostic or therapeutic relevance. Finally, the predominance of activated EM and EM/EMRA subsets in SF-derived CD4+ and CD8+ cells, respectively, across both outcome groups indicates that T cell maturation occurs early at the site of inflammation, regardless of the subsequent clinical course. Collectively, these findings highlight the importance of assessing T cell maturation states both systemically and locally at diagnosis to improve early risk stratification and support personalized disease management in Oligoarthritis.

Treg enrichment has been previously observed in the synovial infiltrate of JIA patients and proposed to play a central role in disease pathogenesis (33–35). Moreover, some studies reported higher Treg frequencies in Oligoarthritis patients who maintained a persistent oligoarticular course compared to those who progressed to the extended form, both locally and systemically, supporting a potential role for Tregs in limiting disease spread (32, 33). The potential of Tregs as a marker for disease extension was assessed by Hunter et al. (37); however, they found no correlation between Treg proportions at onset and clinical course at one year follow-up. Our findings corroborate and extend these observations showing comparable Treg percentages at onset in both SF and PB regardless of clinical course over the two-year follow-up. Taken together, these data suggest that differences in Treg number are unlikely to represent an early mechanism involved in disease progression, rather exerting their regulatory influence later in the disease course possibly in response to chronic inflammation.

Both our group and others have shown that MM enriched in inflamed joints contribute to Oligoarthritis pathogenesis (7, 25, 84, 85) and predominantly polarize toward an inflammatory M1 phenotype in the later disease stages (29, 62). Data presented in this study provide novel insights into the early disease phase, showing that, at onset, MM in SF exhibit a slightly predominant but incomplete M2 polarization independent from the subsequent clinical course. Elevated CD163 expression suggests an early attempt to resolve inflammation (86), while the coexistance of CD80^+^ and CD80^+^/CD163^+^ cell populations indicate a transitional M1/M2 state indicative of a dynamic balance between pro- and anti-inflammatory signals in the joint. These findings extend earlier observations by Hunter et al. (37), showing that SF MM polarization at diagnosis does not differ significantly between patients who would undergo different clinical courses at follow-up, and thus may lack any potential prognostic value. They also align with reports in other chronic inflammatory and autoimmune conditions, which showed coexistence of MM with both pro- and anti-inflammatory phenotypes, or mixed forms shifting polarization over time throughout disease course, supporting the idea that *in vivo* MM polarization represents a continuum of diverse functional states with overlapping or intermediate features (62, 87). Based on current and previous data, we hypothesize that during the early synovial inflammation, infiltrating Mn preferentially adopt a M2-like phenotype (62, 88), potentially preventing excessive immune activation and tissue damage while promoting inflammation resolution (89). However, persistent stimulation may drive a shift toward M1 polarization leading to the amplification of synovitis and chronic inflammation. In PB we also observed highly predominant, albeit incomplete, M2 polarization, characterized by a predominance of CD163+ cells and almost complete absence of CD206 expression. Interestingly, Group 2 patients displayed higher, though not statistically significant, proportions of M1 and mixed M1/M2 populations in PB compared to Group 1, suggesting that M1 skewing may originate in the circulation and later impact the joint environment.

TREM-1, a key member of the immunoregulatory Ig-like receptor family and a major amplifier of MM inflammatory responses (69, 70, 90, 91), has previously been proposed by our group as a driver of M1 reprogramming in the joints of Oligoartritis patients, counteracting M2 polarizing effects of intra-articular hypoxia (62). In this study we observed a significantly higher proportion of TREM1+ cells at onset in both SF and circulating CD14+-gated populations from patients in Group 1 compared to Group 2. Conversely, sTREM1 levels were significantly higher in both SF and PB from Group 2 patients, likely reflecting increased receptor shedding following activation. Taken together with our previous findings (62), this evidence suggest that TREM-1 expression is upregulated on MMs in the early disease stages and its activation by ligands released in response to cartilage/bone damage may promote M1 polarization and sustain the perpetuation and spread of inflammation. Importantly, these findings indicate that the combination of low membrane-bound TREM-1 expression in SFMCs and PBMCs and high sTREM-1 concentrations in SF and PB at disease onset, may serve as potential indicators of the risk for polyarticular extension.

EV proteomic profiling has emerged as a valuable tool for identifying diagnostic and prognostic biomarkers in inflammatory diseases, including JIA (54, 55, 92, 93). Building on previous findings, this study analyzed the immune-related surface antigen profile of EVs isolated from the SF of new-onset Oligoarthritis patients to identify novel prognostic candidate biomarkers using flow cytometry (94–96). SF-derived EVs were specifically selected, as the joints are the primary sites of clinical pathology in Oligoarthritis and SF contains components originating from both local joint tissues and the circulation (38, 55, 97, 98), Consistent with previous studies, SF-derived EVs exhibited both classical EV markers and cell/tissue-specific surface proteins (98). However, the two patient groups showed distinct patterns and/or expression levels of several leukocyte (primarily MM and T cells) and endothelial-associated antigens, suggesting different cell contribution to EVs enrichment in the joint microenvironment. Patients who developed a persistent oligoarticular course displayed higher EV-associated levels of CD3, CD4, CD14, CD2, and CD105, while CD40, CD8, CD86, CD45, CD146, and CD31 antigens were similarly expressed between groups. Notably, HLA-DR was upregulated, and HLA-ABC downregulated, in patients who later extended, suggesting a shift in antigen presentation. Differential expression of integrins and adhesion molecules was also observed. CD29 and CD49e, components of the fibronectin receptor, were more abundant in EVs from Group 1, while the leukocyte adhesion molecule, CD44, from Group 2. All three antigens have been found expressed on immune cells and involved in T cell activation (99) as well as in leukocyte cell recruitment and retention within the joint (100). In addition, CD44 has been linked to synovial inflammation and proposed as a therapeutic target in RA (101), and anti-CD44 antibodies have shown anti-inflammatory effects in murine arthritis models supporting the role of this molecule in sustaining inflammation (100). Importantly, despite the limited sample size, ROC analysis identified EV-associated CD3 and HLA-ABC as strong early indicators of disease course, with AUCs of 0.949 and 0.857, respectively, highlighting their potential value as early prognostic tools. Notably, an inverse relationship was observed between CD3 levels on EVs and the frequency of CD3^+^ cells in SF: higher EV-CD3 expression was detected in patients who maintained a persistent oligoarticular course, while higher CD3^+^ cell percentages were found in those who progressed to the extended form. One possible interpretation is that elevated CD3 on EVs may reflect increased T cell shedding or regulation via EV-mediated mechanisms, potentially serving as a compensatory response to limit local immune activation, while accumulation of CD3^+^ T cells in the joints may reflect ongoing local immune activation, ultimately contributing to disease progression.

Finally, we demonstrated that a combination of cell immunophenotypic and EV markers, specifically the CD3:CD14 ratio, EV-associated HLA-ABC and CD3 levels in SF, along with HLA-DR+ T cell percentages, proportions of naive and EM T cells, and the EM:naive T cell ratio in PB, differed significantly between Group 1 and Group 2 at diagnosis. This composite panel exhibited excellent discriminatory power, achieving an AUC of 1.0 and outperforming any individual marker. These findings suggest that early profiling of such immunomarker panels at diagnosis may provide relevant indications of disease course within two years of follow-up. Notably, the combined immune panels showed a higher discriminatory ability than the combined clinical parameters, highlighting their potential utility for early risk stratification, in particular in patients lacking clear clinical signs of disease extension. However, given the relatively small sample size, we cannot statistically confirm the superiority of the immunologic model, nor conduct a comprehensive multivariate comparison of clinical and biological models to identify the most informative combination. These results should therefore be interpreted with caution, as some degree of overfitting may persist despite the use of internal cross-validation with the GLMNET regularization and LOOCV strategy to minimize this risk. The reported 95% confidence interval for the AUC (0.75–1.00) provides a more realistic estimate of the model performance. It is also important to note that some markers included in the model (e.g., HLA-DR^+^ T cells and EM:naïve T cell ratios) are biologically correlated and may capture overlapping aspects of T cell activation and differentiation. However, Lasso-regularized regression implemented in GLMNET is designed to handle multicollinearity by selecting representative variables from correlated features thereby reducing redundancy (102). Despite these methodological precautions to ensure accuracy, external validation in a larger, independent cohort of new-onset Oligoarthritis patients is essential to confirm the robustness, generalizability, and applicability of these results. If validated, such immunomarker panels could serve as clinically valuable tools for early risk stratification and longitudinal monitoring in Oligoarthritis, either complementing or enhancing current clinical criteria to support more personalized treatment strategies.

Our findings also provide valuable insights into early immune mechanisms underlying disease extension, revealing evidence of both systemic and local immune activation. The detection of PB T cell activation and skewed memory profiles in a clinically joint-limited condition such as oligoarticular JIA is intriguing, suggesting that immune dysregulation is not confined to the initially affected joints, but may reflect early systemic involvement. While Oligoarthritis typically begins as a limited joint disease, up to 40% of patients eventually develop polyarticular extension and 15–30% experience extra-articular manifestations such as uveitis. These clinical observations are consistent with the possibility that systemic immune activation may precede or accompany local synovial inflammation, our findings corroborate previous studies (15, 30) showing that patients who later develop polyarticular disease already display PB immune alterations at diagnosis. The reported presence of elevated systemic inflammatory markers at onset associated with higher risk of disease extension (24, 75) and of overlapping pathogenic T cell subsets in PB and SF in patients with extended JIA (82) further support the presence of a circulating effector T cell pool that sustains inflammation, similar to what observed in RA (103). We extend these observations by showing that increased ESR levels in patients who later developed extended disease are paralleled by specific immune signatures in SF and PB at diagnosis, including increased frequencies of activated T cells, reinforcing the hypothesis that early PB immune activation plays a role in disease progression.

Importantly, identified immune biomarkers, such as TREM-1 expression, T cell activation markers, and EV surface antigens, likely reflect interconnected aspects of early innate and adaptive immunity alterations. Reduced frequency of TREM-1-expressing MM in Group 2 patients at diagnosis may indicate impaired innate immune activation, TREM-1 typically amplifies monocyte- and neutrophil-driven inflammation, and its downregulation may represent a compensatory anti-inflammatory response. Paradoxically, this may hinder resolution of inflammation, enabling adaptive chronic immune stimulation and facilitating disease propagation. At the same time, in fact, patients in Group 2 showed systemic signs of adaptive immune priming at onset, including increased frequencies of HLA-DR^+^ CD4^+^ and CD8^+^ T cells and a higher EM:naïve T cell ratio. These features suggest a predisposition to dysregulated adaptive responses. Interestingly, although total CD4^+^ T cell frequencies were lower in Group 2, their activation status was significantly elevated. This may reflect a shift toward an effector phenotype, or even the acquisition of antigen-presenting functions, as CD4^+^ T cells can acquire MHC class II molecules from professional APC via trogocytosis, potentially propagating immune activation within the synovium (104). The EV data further support this model: reduced levels of CD3 and HLA-ABC (MHC class I) on SF-derived EVs in Group 2 may reflect early T cell dysfunction or exhaustion and impaired antigen presentation, respectively. Together, these findings suggest that early defects in innate immunity (e.g. via reduced TREM-1^+^ monocyte activity), combined with dysregulated adaptive activation, could create a permissive environment for joint-to-joint spread of inflammation. While speculative, our results point to a measurable immune imbalance at diagnosis in patients predisposed to extended disease. Early identification of such patients is crucial to exploit the “window of opportunity”, the critical period immediately after disease onset during which therapeutic intervention may prevent disease progression. As demonstrated in systemic (105) and polyarticular (106) JIA, and proposed for oligoarticular cases (107), early aggressive treatment could improve long-term outcomes by limiting joint damage and spread of inflammation. However, when interpreting immunopathogenic mechanisms, including those underlying disease extension in JIA, it is important to consider that significant differences in cellular composition, phenotypes, and transcriptional programs exist not only between PBMCs and SFMCs, but also with respect to synovial tissue (108). Although some immune cell populations display similar distribution patterns across these compartments, synovial tissue often harbors unique immune and stromal cell states, as well as distinct activation of signaling pathways, that are not fully reflected in SFMCs or PBMCs. For example, while certain cell types, such as CD4+ T cells and myeloid cells, may show partial correlation in abundance between synovial tissue and fluid compartments, these associations are generally limited. Therefore, relying solely on the analysis of blood or SF may fail to capture key aspects of tissue-resident immune pathology.

This study has several important limitations that should be acknowledged. First, the small size of the patient cohort is a common challenge in studies of rare diseases like JIA. The requirement for sample volumes containing adequate numbers of viable cells for flow cytometry further restricted patient inclusion, due to ethical and practical constraints related to the young age of the participants. As a result, although the overall sample size met the threshold established by power analysis, not all immunological parameters could be assessed in all collected specimens, due to limited cell yield, fluid volume, or processing issues. Nevertheless, we ensured a balanced distribution between oligoarticular and polyarticular groups to minimize bias. A second key limitation is the absence of an external validation cohort, which restricts the generalizability of our findings. While the primary analyses were adequately powered, highlighting the potential clinical relevance of immunomarker-based tools for early risk assessment, the biomarkers identified here remain exploratory, as they were derived from a single, small prospective cohort and have not yet undergone validation in independent studies, which is essential before clinical implementation can be considered. Our findings should therefore be interpreted as preliminary evidence, rather than definitive conclusions to address this limitation, we have already initiated a large-scale, multicenter, longitudinal study involving new-onset Oligoarthritis patients with standardized sample collection and two-year clinical follow-up. This study aims to validate and refine the identified biomarkers, assess their generalizability, and explore their potential integration into clinical practice. Longitudinal data will also enable the monitoring of immune profile changes during disease progression and treatment. We will integrate immunophenotyping with clinical and environmental data using multivariate approaches, to determine whether biomarkers are influenced by confounders such as infections, stress or other environmental exposures. Comparative analyses with non-autoimmune arthritides (e.g., infectious forms) are also planned to assess the disease specificity of the identified markers. Given the relevance of sex-based differences in disease pathogenesis, the follow-up study will include sex stratification, which was not feasible in the current analysis due to sample size constraints. Finally, we acknowledge that clinical heterogeneity (e.g. ANA status, age at onset) may affect finding generalizability (10, 23, 76). However, these variables did not differ significantly between outcome groups at onset in our cohort, suggesting limited impact on the results. Treatment variability during follow-up, not accounted for in the current study, may also influence immune profiles. In the validation cohort, we will use multivariable and stratified analyses to assess biomarker performance across subgroups, and apply unsupervised clustering to define immunologically distinct patient subtypes and optimize threshold identification. Assembling a sufficiently powered, clinically homogeneous cohort at disease onset remains a major challenge, representing a long-term effort.

In conclusion, despite its limitations, this study provides a significant contribution toward the identification of early prognostic immune biomarkers and lays the groundwork for a more integrated clinical-immunologic approach to personalized care in oligoarticular JIA.

## Data Availability

The original contributions presented in the study are included in the article/**Supplementary Material**. Further inquiries can be directed to the corresponding author/s.

## Supplementary material

Supplementary Figure 1 Comparative Analysis of MM Polarization Subset Proportions in SF and PB between Patients Undergoing Different Disease Courses. SFMCs (A) and PBMCs (B) were stained with Abs against CD14, CD80, CD206, and CD163 antigens and analyzed by flow cytometry. CD14+ gated cells were analyzed for CD80, CD206 and CD163 expression. Box plots depict the percentages of M1 (CD80+), M2 (CD206 and/or CD163) and M1/M2 mixed populations (CD80+/CD206 or CD80+/CD163) within the CD14-gated population. Results are presented as described in **Figure 1A**. ns, not significant.
