# Supplementary figures and images for: Novel Immune biomarkers for the early stratification of oligoarthritis patients at risk of developing polyarticular extension
Source: Front Immunol. 2025 Oct 8;16:1663663. doi: 10.3389/fimmu.2025.1663663 (PMC12540104; doi:10.3389/fimmu.2025.1663663)

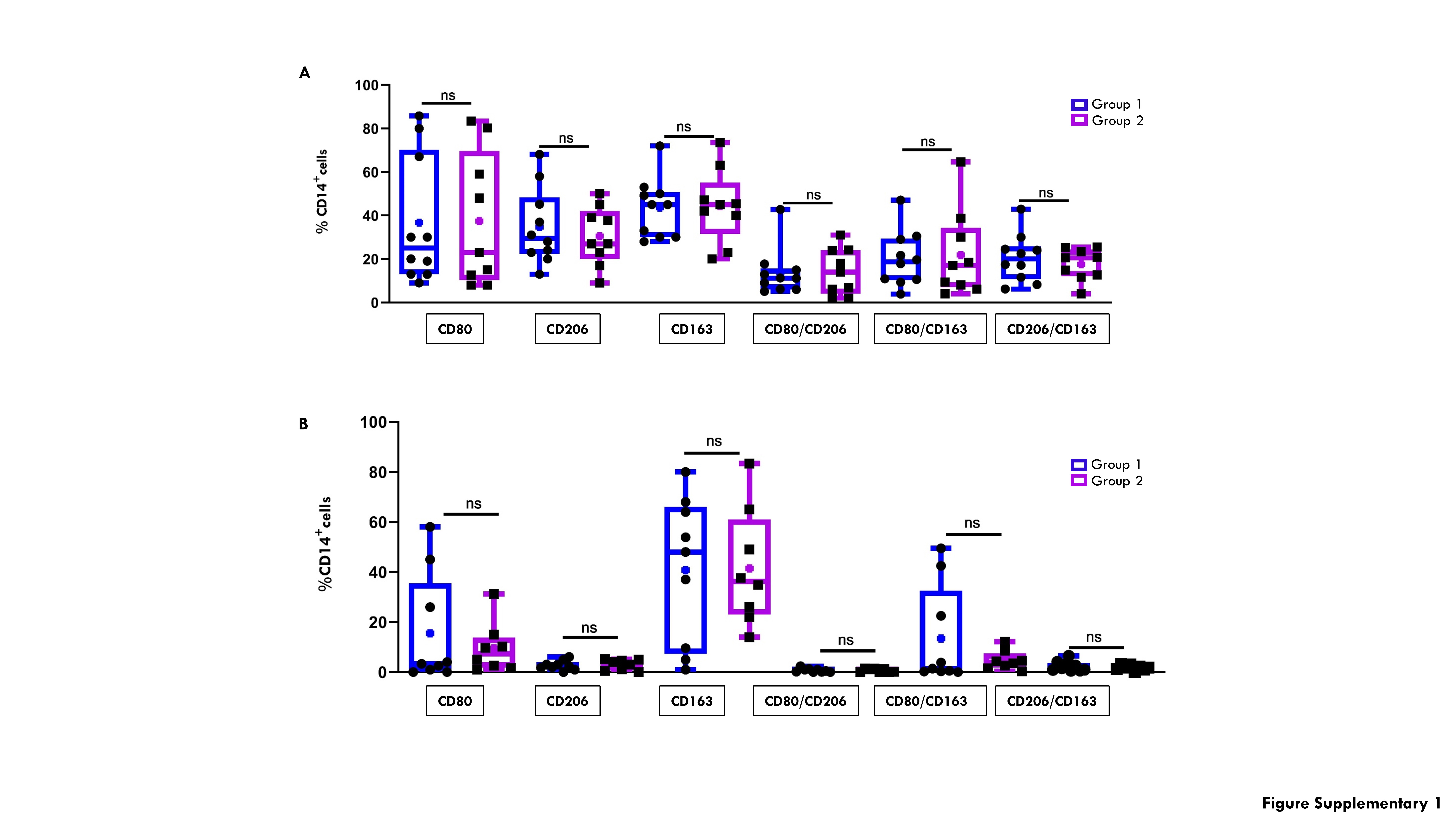

Supplement: Supplementary Figure 1 — Comparative Analysis of MM Polarization Subset Proportions in SF and PB between Patients Undergoing Different Disease Courses. SFMCs (A) and PBMCs (B) were stained with Abs against CD14, CD80, CD206, and CD163 antigens and analyzed by flow cytometry. CD14+ gated cells were analyzed for CD80, CD206 and CD163 expression. Box plots depict the percentages of M1 (CD80+), M2 (CD206 and/or CD163) and M1/M2 mixed populations (CD80+/CD206 or CD80+/CD163) within the CD14-gated population. Results are presented as described in Figure 1A . ns, not significant. [file Image1.jpeg]
